# Factors influencing the integration of a palliative approach in intensive care units: a systematic mixed-methods review

**DOI:** 10.1186/s12904-020-00616-y

**Published:** 2020-07-22

**Authors:** Hanan Hamdan Alshehri, Sepideh Olausson, Joakim Öhlén, Axel Wolf

**Affiliations:** 1 Institute of Health and Care Sciences, Sahlgrenska Academy, University of Gothenburg, Gothenburg, Sweden; 2 Princess Nourah bint Abdulrahman University, Riyadh, Saudi Arabia; 3 Institute of Health and Care Sciences and University of Gothenburg Centre for Person-Centred Care, Sahlgrenska Academy at the University of Gothenburg, Gothenburg, Sweden; 4 Palliative Centre, Sahlgrenska University Hospital Region Västra Götaland, Gothenburg, Sweden; 5 Department of Anaesthesiology and Intensive Care Medicine, Region Västra Götaland, Sahlgrenska University Hospital/Östra, Gothenburg, Sweden

**Keywords:** Palliative care, Critical care, Implementation science, Intensive care units, Review

## Abstract

**Background:**

While a palliative approach is generally perceived to be an integral part of the intensive care unit (ICU), the provision of palliative care in this setting is challenging. This review aims to identify factors (barriers and facilitators) influencing a palliative approach in intensive care settings, as perceived by health care professionals.

**Method:**

A systematic mixed-methods review was conducted. Multiple electronic databases were used, and the following search terms were utilized: implementation, palliative care, and intensive care unit. In total, 1843 articles were screened, of which 24 met the research inclusion/exclusion criteria. A thematic synthesis method was used for both qualitative and quantitative studies.

**Results:**

Four key prerequisite factors were identified: (a) organizational structure in facilitating policies, unappropriated resources, multi-disciplinary team involvement, and knowledge and skills; (b) work environment, including physical and psychosocial factors; (c) interpersonal factors/barriers, including family and patients’ involvement in communication and participation; and (d) decision-making, e.g., decision and transition, goal conflict, multidisciplinary team communication, and prognostication.

**Conclusion:**

Factors hindering the integration of a palliative approach in an intensive care context constitute a complex interplay among organizational structure, the care environment and clinicians’ perceptions and attitudes. While patient and family involvement was identified as an important facilitator of palliative care, it was also recognized as a barrier for clinicians due to challenges in shared goal setting and communication.

## Background

Intensive care units (ICUs) are specialized hospital wards where seriously ill patients, often with life-threatening conditions, are treated by specialized care professionals and receive intensive care [1], with the essential aim of providing life-saving and life-sustaining care [2, 3]. While mortality rates have drastically improved in recent decades [4], a substantial proportion of patients receive intensive care who will either not survive or who will be discharged for end-of-life care, often close to death [5]. This highlights the relevance of integrating a palliative approach into the care process in intensive care settings. Thus, during recent decades, the point of departure of critically ill patients within the ICU has changed, shifting towards more chronically ill patients who deteriorate in their chronic condition. The majority of patients in the ICU are elderly, representing a vulnerable population with a history of chronic and often comorbid diseases such as heart failure or COPD. The COVID-19 pandemic shows this very clearly, as many elderly patients with chronic diseases are admitted to the ICU due to respiratory failure [6, 7]. Clearly, the COVID-19 pandemic necessitates the inclusion of responses with a palliative approach, such as active symptom relief, communication and recognition of dying. When chronic conditions lead to deterioration and occur among older adults, the question of the integration of a palliative approach to patient comfort and end-of-life care intensifies.

In general, a palliative approach aims to relieve suffering for patients with life-limiting conditions and for those who are dying and to manage symptoms, increase the level of care comfort and provide support to family members [8, 9]. Despite increasing awareness of integrating a palliative care approach in the ICU, there are challenges, given the somewhat contradictory aims of intensive care and palliative care, i.e., in providing life-saving treatments vs treating dying as a normal process. However, studies have shown the benefits of integrating palliative care into intensive care [10–13], for example, in relieving distress for patients and their families during end-of-life care. Nevertheless, a review by Kahveci [14] on attitudes and beliefs pertaining to integrating a palliative approach in the ICU shows that there are many challenges, such as sociocultural factors, legal regulations and a lack of awareness of a palliative approach. The integration of a palliative approach has been described as challenged by a lack of resources for symptom management and cultural and societal values and beliefs about death and dying [15]; moreover, structural barriers (for example, limited specialties and resources) appear especially difficult to change [16].

To date, no mixed-methods review has been conducted that integrates both quantitative and qualitative evidence to frame a broader picture of factors that influence the adoption of a palliative care approach in the ICU. Hence, there is a need to synthesize the findings of quantitative and qualitative research studies on the factors (facilitators and barriers) influencing a palliative approach in the ICU from the perspective of allied health professionals. In particular, there is a need to increase knowledge of contextual factors (attitudes, perceptions and structural/organizational) influencing this integration of care perspectives, which could be considered to be exclusively related to each other.

### Aim

To identify factors influencing a palliative approach in intensive care units, as perceived by health professionals.

## Methods

A mixed-methods systematic review was undertaken with the aim of identifying, assessing, analysing and synthesising the current research findings [17]. The first step in the process was to perform a review protocol (registered in the international prospective register of systematic reviews (PROSPERO) (CRD42018099786). Second, systematic literature searches were conducted, and relevant literature was selected. Third, we performed quality assessments of the included articles [18]. Finally, we analysed and synthesised the articles’ findings, taking the assessed quality into consideration.

### Eligibility criteria and review selection

A search guide was developed based on the research concepts and questions within the inclusion and exclusion criteria. The authors used a PEO framework (population, exposure, outcome) [17] and focused on the following:

- **P**opulation – allied health care professionals in the ICU;
- **E**xposure – integration or implementation of a palliative approach;
- **O**utcome – factors (facilitators and barriers) influencing a palliative approach; and.
- Context – ICU.

Two expert medical librarians supported our search process.

The inclusion criteria were as follows:

1. Studies focusing on factors influencing the integration of a palliative approach for adult patients admitted to the ICU;
2. Studies highlighting health care professionals’ experiences or perceptions of the integration or implementation of palliative care in ICUs;
3. Studies written in the English language; and.
4. Peer-reviewed studies published between January 2007 and January 2018.

Our exclusion criteria were as follows:

1. Non-empirical studies (e.g., editorials, brief reports);
2. Studies in paediatric and neonatal intensive care;
3. Studies reporting the frequency of palliative care in the ICU and the effect of palliative care on the mortality rate or length of stay in the ICU; and
4. Studies regarding palliative care policy.

### Literature search strategy

In this review, we have chosen to use broad concepts and surrogate words and associated definitions. We used the search terms targeting the integration of a palliative approach, such as implementation, palliative care, and intensive care units, as well as synonyms for these terms (see Additional file 1). The university librarian at the University of Gothenburg performed an electronic database search of the following databases: AMED, PubMed, EMBASE, PsycInfo, Sociological Abstracts, Web of Science, Scopus and Cinahl. In total, 1843 citations were identified (AMED *n* = 21, PubMed *n* = 507, EMBASE *n* = 376, PsycInfo *n* = 46, Sociological Abstracts n = 3, Web of Science *n* = 160, Scopus *n* = 495, and Cinahl *n* = 235).

### Selection of articles

**Fig. 1:**
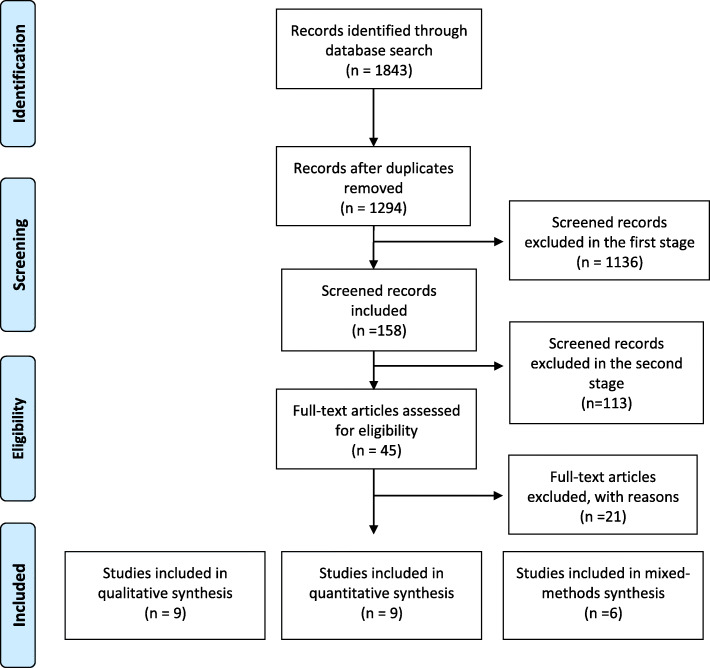
PRISMA flowchart

In total, 1843 articles were identified in the initial search, of which 462 were duplicates and were thus deleted before we imported the remaining 1407 articles into an web based systematic reviews software for blind screening (Rayyan QCRI, developed by Qatar Computing Research Institute) (see Fig. 1). Two authors (HH, AW) performed blind screening of the articles in Rayyan following the inclusion and exclusion criteria (above). In the first round, the article titles and abstracts were screened (in the online tool Rayyan). In the second round, eligibility was assessed based on the blind reading of 158 full-text articles by two authors (HH, AW). The authors summarized and documented the reasons for inclusion and exclusion. In the third round, which was unblinded, two authors (HH, AW) compared and discussed the relevant articles, and if there was disagreement, consensus for final inclusion was achieved by consulting all members of the research team. As a result, 24 articles were ultimately included in this systematic review (see Fig. 1).

### Quality assessment

Depending on the type of study, the quality assessment was carried out as follows: nine qualitative studies and one quantitative study with an RCT design were evaluated using the Critical Appraisal Skills Program (CASP). The remaining quantitative studies (*n* = 8) were evaluated using the Best Evidence topics (Best BETs) critical appraisal checklists for survey study design. The Mixed-Methods Appraisal Tool (MMAT) was used to evaluate the studies using a mixed-methods approach (*n* = 6). The researchers evaluated each of the studies and developed scores for all types of tools. For the quality assessment summary (see Additional file 2).

### Analysis and synthesis

A thematic synthesis approach was selected for the study. Thematic analysis has been used in mixed-methods systematic reviews that address questions such as identifying barriers or facilitators from evidence and identifying patterns within the findings [17]. While thematic synthesis is generally used for the synthesis of qualitative studies, it may also be useful when there is heterogeneity in outcome variables and measurement in quantitative studies. Before the beginning of the analysis stages, the first author (HH) read each included article several times, data were extracted, and quality assessments were performed for quantitative and qualitative analyses separately. A three-stage thematic analysis process was undertaken [18]. In the first stage, the first author (HH) focused on factors influencing a palliative approach in the ICU based on results from quantitative data. In the second stage, we focused on factors influencing a palliative approach in the ICU based on results from qualitative data. In the third stage, two authors (HH, AW) integrated the analyses using thematic synthesis with a focus on factors influencing a palliative approach in the ICU based on results from both quantitative and qualitative data. They also repeatedly checked the analysis against the articles and started to thematize the findings into influencing factors. Inconsistencies in preliminary analysis were discussed, and the other authors were consulted (SO, JÖ). In this stage, we sought to distinguish the influencing factors into overarching analytical categories (type of factors) and more descriptive categories (specific factors). The similarities and differences between the findings were highlighted and grouped to finalise the development of descriptive categories (factors) and analytical categories (type of factors). All authors were involved in reviewing and checking the accuracy of the final findings.

## Results

Study characteristics and quality assessment summary: Fig. 1 shows a standard flow chart reporting the results of the bibliographic search and screening (it follows the guidance of the Preferred Reporting Items of Systematic Reviews and Meta-Analyses (PRISMA). In total, 24 studies were eligible for the review: nine qualitative, nine quantitative and six mixed-methods studies. In total, the articles reported studies with 2545 participating allied health professionals. Studies were conducted in ten countries worldwide, with most studies conducted in the United States of America (*n* = 11), the United Kingdom (*n* = 5), the Netherlands (2), Germany (2), Australia (1), Canada (1), Japan (1) and Brazil (1). A summary of all included articles and their quality assessments are shown in (see Tables 1, 2, and 3). In total, eight articles were assessed as having either moderate-to-high or high quality, eight articles were assessed as having moderate quality, four articles were assessed as having low-to-moderate quality, and four articles were assessed as having low quality (reference [33, 34, 38, 40]) (see additional file 2)*.*

**Table 1:** Data extraction and quality assessment of included articles divided into qualitative articles

First author, publication year, country	Aim of the study	Participants	Methodology/data gathering	Main outcome	Barriers	Facilitators	Quality assessment
Zomorodi [19] 2010/USA	The aim of the study was to explore nurses’ definitions of quality EOL care and to describe the activities that promote quality EOL care in the ICU	Nurses (*n* = 9)	Interviewed	The interview indicated that nurses undertook a number of methods to improve the quality of EOL care available. Expressions such as ‘balancing’, ‘trial and error’, ‘taking a step back’ and ‘coaching the physicians’ were mentioned. These, combined with a number of personal, environmental and interrelationship factors, were found to both enhance and limit the ability of critical care nurses to administer EOL quality.	*Moral distress caused by providing end of life care. *Noise and technology *Lack of time and education *Fragmentation of care *Neglected transformation of care from curative to palliative	*Trial and error in daily practice	Moderate-high
Anderson [20] 2015/USA	To determine the perspectives of key stakeholders regarding how prognostic information should be conveyed in critical illness	Critical care clinicians (*n* = 45) Surrogates (*n* = 47) Experts in health communication (*n* = 26)	In-depth semi-structured interviews	It was generally agreed by surrogates that there was a need for open, completely truthful prognosis to be made available to the family. In particular, it was stated that emotional support should be attuned to their needs and that it should be ensured that prognosis was fully understood. A further recommendation was that, in addition to making predictions for the patient, clinicians should make available comparable statistics, such as radiographic images, explaining the physical symptoms of the disease. This recommendation was not endorsed by the majority of physicians, who considered that numerical statistics were not constructive in such cases. This contrasted with the opinions of surrogates and physicians from other disciplines who considered their usage beneficial. Physicians were urged to raise the subject of death early on, when patients were committed to the ICU, and to amplify it with greater detail as the clinical situation progressed. The disclosure procedure should be initiated by physicians, after which there is a need for the various members of the team involved to substantiate the physician’s prognosis to prepare the family for the ultimate outcome.	*ICU culture *Ineffective communication with families *Inadequate staffing and time *Lack of availability of space *Lack of clinician training in communication *Lack of explanations regarding medical terminology	*Use different type of material for medical information *Prognostication information model *Engage different disciplines	Moderate
Liaschenko.,J [21] 2009/USA	To investigate factors influencing critical care nurses’ provision of end-of-life care and their inclusion of families in that care	Nurses (*n* = 27)	A focus group interview	“Supporting Families’ Dying Process” clarifies how critical care nurses organize information to construct the ‘big picture’ of the patients’ deteriorating status and artfully communicate this to families	*Acting on information *Trust and knowledge *Time challenges *The culture of the unit	*Support family involvement and communication	Moderate
Baggs [22] 2007/USA	To study limitations of treatment decision making in real time and to evaluate similarities and differences in the cultural contexts of 4 ICUs and the relationship of those contexts to end of life care decision making (EOLDM)	Health care providers, patient and families (*n* = 130)	Ethnographic/observation and interviews	It was agreed that ICUs were not monolithic. There were similarities, but important disparities in EOLDM were recognized in the formal and informal guidelines, technology significance and utilization, physician roles and interactions, procedures such as unit rounds, and timing of EOLDM initiation.		*Planned family meetings	Moderate
Ranse.K [23] 2012/Australia	To explore the end-of-life care beliefs and practices of intensive care nurses.	Nurses (n = 5)	A descriptive exploratory qualitative/a semi-structured interview	Three main categories were identified: end-of-life care beliefs, end-of-life care in the context of ICU and end-of-life care facilitation. Factors that contribute to end-of-life care experiences and methods of intensive care nurses were integrated into the first two classifications.	Lack of advanced practice skills *Lack of understanding of the complexity of palliative care *Ambiguity of palliative care *Limited support (protocol) *ICU culture *Lack of emotional support	*Family involvement in comfort care environmental peaceful setting (single room) *Creating an atmosphere *Changing environment	Moderate –high
Radcliffe, C [24] 2015/UK	Describes the views of health professionals using a supportive care pathway in intensive care	Healthcare provider (*n* = 10)	Semi-structured interviews	The effects of the supportive care pathway on patient care were endorsed by most respondents. It was considered particularly effective in allowing agreement on care planning. Doubts were expressed in identifying the right patients for the pathway, which was mentioned as being a euphemism for dying.	*Difficulty in identifying care pathway *Lack of symptom control *Autonomy of clinicians in using supportive care *Pressures and priority of work	*Enabling consensus in care planning *Supportive care pathway framework *Involving nurse in decision making	Moderate –high
Gulini [25] 2017/Brazil	To learn the perception of health professionals in an intensive care unit towards palliative care	Nurses (n = 12), nursing technicians (n = 11), physical therapists (n = 5), doctors (n = 9)	Semi-structured interviews.	The survey highlighted the need for care to be executed at the final stage of life in order to avoid unnecessary actions, emphasis to be placed on comfort care and the need for a better care system and increased team training.	*Lack of standardized care *Lack of team training *Ineffective communication		Moderate-high
Walker [26] 2010/UK	To explore doctors’ and nurses’ experiences of the impact of the LCP in two intensive care units	Nurses (*n* = 6)	Interview	The nurses’ experience of the LCP depended on their responsibilities and was based on frequency of use and educational level received in the LCP. Education and adequate support have been recognized as being fundamental to the effective implementation of palliative care approaches.	*Doctors struggle with palliative care decisions*Lack of guidance regarding applying palliative care *Lack of protocols for palliative care in ICU *Lack of familiarity with palliative care	*Palliative care guidelines	Moderate
Holms.et al. 2014/UK [27]	To explore the experiences of ICU nurses who had provided EOLC to patients and their families	Nurses (n = 5)	A phenomenon- logical/Semi structured interviews	Five themes were identified: use of integrated care systems, communication, the environment, education and training, staff distress. Nurses stated that they were unprepared to provide end-of-life care. Inadequate communication between staff, patients and families while providing care for patients at end of life.	*ICU environment affecting palliative care *Lack of education and training in palliative care *Communication and decision conflict *Staff distress	*Integrated care system *Staff ratios	Low-moderate

**Table 2:** Data extraction and quality assessment of included articles divided into quantitative method articles

First author, publication years, country	Aim of the study	Participants	Methodology/data gathering	Main outcome	Barriers	Facilitators	Quality assessment
Latour, J [28] 2009/Netherlands	To investigate experiences and attitudes of European intensive care nurses regarding end-of-life care (EOL)	Nurse (*n* = 419)	A self-administered questionnaire	Approximately three-quarters of respondents (73.4%) said they took an active part in decision-making, while a slightly larger percentage (78.6%) stated their commitment to EOL decisions being made with family involvement. Just under half of the respondents stated that patients should be kept deeply sedated, almost equal numbers were for and against the maintenance of nutritional support (41.6 and 42.3%, respectively). The general consensus was against patients being moved to single rooms (78%).		*Presence of family members during dying process	Low-Moderate
Jox, R [29] 2010/Germany	To investigate the practices and perspectives of German intensive care nurses and physicians on limiting LST.	Nurses (*n* = 268) and physicians (*n* = 95)	Survey/questionnaire written in German	It was apparent that half of the junior physicians and an equal number of nurses were unclear as to the correct decision-making process. The physicians were the least certain about the procedures, citing a lack of training, and were worried about the possibility of litigation if there had been a failure in carrying out the correct procedure	*Decision-making process *Lack of standardized documentation practice *Ignoring involved nurses and family in decision-making		Moderate
Voigt [30] 2015/USA	To evaluate the frequency, characteristics, and outcomes of ethics consultations in critically ill patients with cancer.	Nurse ethicists (*n* = 30) Nurses with 10 years of experience (*n* = 46) Team (*n* = 50) Patients (*n* = 35)	Retrospective analysis of all adult patients with cancer	Ethics consultations between the ICU team and the patients or their surrogates arose from a lack of agreement between the parties on EOL care. This was evident from a study of 53 patients, most of whom had surrogates, and two-thirds of whom lacked decision-making ability.	*Conflict between the patient’s caregiver and the ICU team *Conflict between physicians about palliative care	*Ethics consultations	Moderate- High
Crump SK [31] 2010/USA	To explore the obstacles (barriers) to, and supports for, EOL care in their critical care units	Nurses (*n* = 56)	Survey/questionnaire	The outcome was as follows: (1) families and patients needed clear data to make EOL choices; (2) physician-related problems influence the capacity of nurses to provide quality EOL care; (3) critical care nurses need more expertise, skills and a sense of cultural competence to deliver quality care; and (4) clear advance directive guidelines can decrease confusion about care objectives.	*Different family and friends issues *Continuation of aggressive care *Lack of patient and family knowledge about prognosis	*Designate one contact person per patient *Teach family members about how to deal with the process of dying *Meeting of doctor and family after death	Low -Moderate
Kirchhoff, K [32] 2010/USA	To describe the training, guidance, and support related to withdrawal of life support received by nurses in intensive care unit	Nurses (*n* = 475)	Survey/questionnaire	From respondents’ responses (48.4%), almost half of the nurses in the survey, it was evident that only a small part of their basic education related to the withdrawal of life-support systems (15.5%). In addition, two-thirds of those questioned received no further on-site training (63.1%). The evidence indicated that nurses’ actions during withdrawal were directed primarily by the overseeing physician’s instructions (63.8%), thereafter, by their following a standardized care plan (20%) and finally by complying with standing orders (11.8%).	*Lack of training from work site *Procedural difficulties *Individual physician’s orders *Lack of a palliative care education programme *Shortage of staff (workload) * Insufficient emotional support for nurses	*Nurses involvement in family meeting	High
Schimmer [33] 2011/Germany	To assesses the medical and ethical criteria and the method of WH and/or WD of life support treatment in cardiac intensive care units	Clinical director, senior ICU physician and head nurse (*n* = 237)	Survey/questionnaires	The three principal reasons for withholding/withdrawing (WH/WD) life-sustaining treatment were given as cranial computed tomography (CCT) with poor prognosis (91.9%), multi-organ failure (70.9%) and failure to assist device therapy (69.8%). A third (32.6%) of respondents admitted that their decision-making was ethically influenced. The perceptions of the three professional groups in the survey differed considerably over the decision-making issues: multi-organ failure (*P* = 0.018), failure of assist device therapy (*P* = 0.009) and cardiac index (P = 0.009). The ethical issues were the poor expected quality of life (*P* = 0.001), the patient wanting to limit medical care (*P* = 0.002), interoperative course (*P* = 0.054), and the family choice (*P* = 0.032).	*Ethical aspects influence the decision-making process		Low
Kamel, G [34] 2015/USA	To identify residents’ knowledge and their perceived barriers of PC-end-of-life (EOL) care utilization in the ICU	Residents (n = 30)	Cross-sectional study	Residents stated that the greatest failure was in the goals of care provided by the medical teams and those expected by the patients and their families, cited by 18.7% of respondents. The patient was required to be terminally ill before a successful palliative care consultation could be obtained (22.9%).	¨*Discrepancies in care goals between the medical team and patients/families *Lack of advanced directives at the time of admission *Lack of a specific protocol for palliative care	*Specialized palliative care team	Low
Friedenberg [35] 2012/USA	Identifying perceived barriers to optimal EOL care based on level of physician training or by discipline	Residents (*n* = 125) Fellows (*n* = 20) Attendants (*n* = 13) Nurses (*n* = 60)	Survey/questionnaire	There were important variations in reported obstacles to EOL care by training and education level, discipline, and organization, especially in the field of education and training. A lower percentage of residents (20%) revealed insufficient training in EOL care and as a big or enormous obstacle to providing palliative care than attendants (62%), fellows (55%) or nurses (36%). Communication related to language difficulties (*p* = 0.008) and insufficient training in pain and anxiety recognition (*p* = 0.001) were identified as barriers to providing EOL reported by nurses.	*Difficulty communicating due to language barriers *Lack of advance directives *Inadequate care goals *Inadequate training in recognition of pain and anxiety *Patient’s inability to participate in care goals		Moderate- High
Noome, M. [36] 2016/Netherlands	To examine the effectiveness of supporting intensive care units in implementing the guidelines	Nurses (*n* = 153) Intervention group Nurses (*n* = 112) Control group Family (*n* = 18) intervention group Family (*n* = 15) control group	RCT intervention group	Results showed that the intervention group followed the guidelines in most aspects more closely than the control group. Families reported that the intervention group showed a markedly higher level of patient care and general nursing care in comparison to the control group.	*Lack of social worker support *Time constraints in implementing strategies *Healthcare provider resistance to change	*Involving family in patient care *Involving other professionals, *Education and support for professionals and the use of guidelines * Team meeting * Providing managerial support for implementing guidelines	Moderate- High

**Table 3:** Data extraction and quality assessment of included articles divided into mixed-methods articles

First author, publication years, country	Aim of the study	Participants	Methodology/data gathering	Main outcome	Barriers	Facilitators	Quality assessment
Hansen, L [37] 2009/Portland USA	To examine how use of multiple interventions could improve nurses’ experience of end-of-life care.	Phase 1 nurses (*n* = 91) Phase 2 nurses (*n* = 127)	Questionnaire: a 5-subscale tool consisting of 30 items scored on a Likert scale Qualitative data open-ended questions	In general, scores on the five subscales were exceeded, with the levels of nurse perception improving over time, particularly during the second stage when the scores were greater than the set criteria. It was evident that the pace of some improvements was consistent across units, whereas others were implemented at different times to reach the overall mean score.	*Lack of written symptom control protocol * Insufficient communication among nurses, physicians, and patients’ families *Lack of spiritual care *Physicians’ behaviours, influence palliative care	*Bereavement programme	Moderate
Centofanti.et al. [38] 2016/Canada	To describe residents’ experiences with end-of-life (EOL) education during a rotation in the intensive care unit (ICU) and to understand the possible influence of the 3 Wishes Project.	Residents (*n* = 33)	Mixed-methods Semi- structured interviews	It was evident that there were three major issues. (1) Training is paramount in the care of EOL patients as a death in the intensive care unit (ICU) can create a feeling of helplessness, especially as it is difficult to form an empathetic relationship with dying patients. In particular, it is considered that there is not enough EOL training, the very quality that is valued by patients. (2) The project re-emphasizes the elements of dying, focusing more on the humanity of the practice, giving prominence to the family’s involvement, encouraging a higher level of emotional interaction and ensuring that the care is an ongoing process during and after the patient’s death. (3) Encouraging EOL dialogue and reflection, assisting residents to react in a palpable manner and employing role modelling allows the project to subscribe to experimental education.	*Difficulties in communication with dying patients related to ICU culture * Inadequate education and palliative care skills	*Facilitate palliative care dialogue *Facilitate family engagement learning	Low
Anderson WG [39] 2017/USA	To implement and evaluate a palliative care professional development programme for ICU bedside nurses.	Nurses (*n* = 428) Nurse leaders (n = 8)	Mixed methods A survey was completed by bedside nurses Qualitative data notes taken by nurses’ leaders	It was encouraging to learn that nurses assessed their EOL skill level to be significantly higher post-workshop; they identified 15 tasks such as making sure the family fully understood the situation when convening a family meeting and helping to alleviate family distress (*P* < 0.01 vs. pre-workshop) care needs.	*Lack of palliative care team in ICU *ICU is a noisy environment	*Palliative care nursing programme (hospital setting) *Involve multi-specialty *Palliative care nursing instructor *Communication workshop	Moderate
Wysham N. [40] 2017/UK	To explore attitudes about ICU-based palliative care delivery, preferred screening practices for finding appropriate recipients of specialist consultation, and triggers themselves	Nurses (*n* = 150) Intensivist (*n* = 114) physicians (*n* = 39)	Survey included open-ended questions	Three-quarters of the 225 cases reviewed stated that palliative care consultation was inadequate. The favoured method was selecting those eligible by electronic health record identification searches for specialist consultation. From 123 cases (41%), only 6% (in this instance 17 cases), considered that the present system was sufficient. Metastatic malignancy, EOL decision making, persistent organ failure and non-realizable care aims were the most identifiable triggers for consultation.	*Absence of palliative care consultation in ICU *Unrealistic goals of care, end of life decision making, and persistent organ failure.		Low
Satomi Kinoshita [41] 2007/Japan	Examine why intensive care unit (ICU) nurses experience difficulties in respecting the wishes of patients in end-of-life care in Japan	Nurses (*n* = 1158)	Survey/questionnaire Interviews	The reasons were compounded, as decision making was often conducted by those who had no concept of patient wishes, even those which were often unachievable, where the death was sudden and constrained by time. It was identified that the majority of nurses sought to fulfil the wishes of a dying patient. Their manner of death in the ICU left ethical questions to be answered. However, nurses appreciated that to honour a patient’s last wishes in such a situation was frequently impracticable. It was evident from the results of the investigation that there was a lack of meaningful discussion on how to respect the wishes of dying patients.	*Inability to respect patient’s wishes *Excessive treatment in the role of the ICU *ICU environment is inappropriate for dying *Rapid deterioration and sudden death *Lack of information (patients’ wishes) and patients’ family		Low-moderate
Zib, M [42] 2007/UK	This pilot audit addresses the feasibility of developing an end-of-life (EOL) decision making audit and quality improvement tool and applying it in the intensive care setting	Patients records (*n* = 47) Intensivists (n = 15)	Charts were audited Structured interview with the intensivist	Over half of ICU deaths (55%) followed the withdrawal of treatment. The vast majority of reasons for withdrawal were given as futility or treatment failure. There were no recorded instances of dissension between the family and the medical staff. Critical care physicians, the intensivists, had a high level of credence in making EOL decisions.	*Treatment failure or futility was the reason cited for withdrawal. *Confidence among intensivists Strong support for advance planning and for audit of EOL decision making was highlighted.	*Consultation with ICU colleagues was rated as the most helpful factor in decision making. *Intensivists wished for earlier and more active support from the admitting medical officers in decision making.	Moderate

### Influencing factors (facilities and barriers)

Four types of influencing factors were identified: (1) organizational structures, (2) working environment, (3) patient and family involvement, and (4) palliative care decision-making. We present the summary of these types of influencing factors below and specific factors with related facilitators and barriers in Table 4 Below, each of the four influencing factors are presented with specific facilitating and hindering factors.

**Table 4:** Influencing factors of the palliative care approach in the ICU

Type of influencing factors	Specific factors	Barriers	Facilities
**1. Organizational structures**	Management resources	- Lack of time, resources and staff shortages to care for palliative patient in ICU [19, 34]. - Simultaneous requirement to care for other patients while staff work with patients’ palliative need [32]. - Lack of time to develop and implement strategies [36]. - Lack of training and education as a result of time and resource constraints [19, 41]. - Lack of spiritual support (assistance is unavailable at weekends) [37, 42]. - Inadequate support of junior nurses from team leaders [23]. - Absence of palliative care physicians and senior nursing staff in relation to advance directive (AD), orders in ICU and difficulties within the palliative care process [32].	- Staff ratios. For example, in ICU nurses work in patient/nurse ratios of either one to one or one to two. This allows time to be devoted to dying patients [27]. - Assign a nurse for the patient in late palliative stage, for example, patient and family should be cared for by a nurse who is known to them [28]. - A bereavement programme to support patients and families (use bereavement material) [37]. - Facilitating palliative care dialogue (standardized tools) [38].
	Policies and guidelines	- Lack of protocol and policies guiding palliative care in ICU [22, 23, 34]. - Lack of written protocol for palliative care nursing such as pain management, dyspnoea, etc. [37]. - Doctor resistance to applying policy [37, 38]. - Physicians unfamiliar with the guidelines and resistant to using them, plus difficulties encountered in the removal of all mentors [26]. - Insufficient standardization of care [25] and the presence of procedural difficulties [32].	- Strong leadership and management team support (supportive factors) [36]. - Guideline recommendations regarding both direct care for palliative patients and palliative care decisions [26]. - employing guidelines and care policy designed for a humanistic approach for example, medication guidelines designed to improve symptom control [26]. - An integrated care system (clear guidance, reduced paperwork, adequate structure) [27]. - Formulating the physician’s strategy [19]. - Open visiting times for family and friends [28].
	Knowledge and skills	- Inadequate education and knowledge of palliative care among nursing staff responsible for delivering care to patients in ICU [21, 38]. - Inadequate training for physicians and nurses regarding communication skills [35]. - Lack of understanding about the complexities involved in providing palliative care in ICU [23]. - Lack of requisite knowledge, skills and experience among physicians [37, 38]. - Lack of skill in the provision of care for dying patients [23]. - Inadequate information relating to palliative care issues within both current nursing curriculums or courses and hospital orientation [32]. - Insufficient preparation for palliative care decision-making (inadequate professional training) [29, 32]. - Unpreparedness of ICU nurses for shifting from curative to palliative care models [19].	- Involving training teams in specialized palliative care while providing care for patients in ICU [25]. - Hospital training and the development of a palliative nursing programme [39]. - Specialist palliative nursing coaching [39]. - Training workshop communication programme for bedside nurses [39]. - Trial and error are useful ways to learn from nurses’ experiences [19]. - Education and professional support while implementing improvements to guidelines [36].
	Multidisciplinary team involvement	- Lack of nursing staff involvement in palliative care decision making [28, 29, 42]. - Lack of palliative care team integration within the ICU [37, 40].	- Involvement of stakeholders from different levels and specialties [39]. - Direct palliative care team involvement in care for patients in ICU [34].
**2- Working environment**	Physical environment	- Absence of infrastructure in the ICU to facilitate family involvement in palliative care (insufficient space for meetings) [20, 25, 35]. - Inadequate organizational support in promoting humanistic environment in ICU [23] - The challenging, hectic and noisy nature of ICU culture [20, 21, 37, 38]. - Inappropriate environments for dying patients [27, 41].	- Modified bedside environment and use single room for dying patients [23, 36].
	Psychosocial environment	- Moral distress acts as barrier to providing palliative care in the ICU [19]. - Distress experienced by nursing staff due to lack of help from managers [27]. - Colleagues’ unwillingness to appreciate the complexity of palliative care (staff compliance with changes) [23, 36]. - Insufficient emotional support for nurses during and after their providing palliative care for patients in ICU [27, 32].	
**3-Patient and Family involvement**	Conflict	- Disagreements, unwillingness to discuss them and conflict between families and physicians regarding palliative care process [22, 23, 32, 35]. - Family’s refusal of care on the grounds of religious belief [35].	- To tailor and adapt the object of care in collaboration with the patients and families [22].
	Participation	Lack of family involvement in any documented wishes that have been expressed by the patient [42]. - Language and culture barriers relating to patients and/or their families [35]. - Lack of understanding and education among patients and family concerning the prognosis and the continuity of palliative care [22, 31]. - Patient inability to participate in palliative care decision-making [35]. - Patient’s wishes regarding palliative care were insufficiently documented prior to their admission to ICU [42].	- Family participation and involvement in patient care and decision-making (family-centred care [23, 28, 36, 38]. - Patient and family wishes considered prior to actual decision-making [38]. - The establishment of a patient advocate and medical translation team by nurses involved in family meetings [32]. - Respect for patients’ wishes [38].
	Information/ communication	- Lack of effective communication with family members [20, 34]. - Family’s requests for updates on patient’s prognosis [31]. - Insufficient information provided to patients and families about death [41].	- Using multiple means to communicate medical information regarding patients’ prognoses to their families [20]. - The allocation of a single point of contact for all family members [31].
4**. Palliative Care Decision-Making**	Transition of care objectives	Fragmentation of care objectives by different physicians [19]. - Discrepancies between the care objectives of the medical team and the family [34]. -Disagreement between team members about comfort care decisions and inconsistencies in palliative care [27, 36]. - Continuation of aggressive and life-supporting treatments [31, 41]. - Absence of a care plan for palliative care [31]. - Inconsistent attitudes, approaches and beliefs among physicians providing palliative care [37, 42]. - ICU patient decision-making potentially negated by incapacity [41].	- Clear and defined goals for providing comfort and care [24]. - Consensus regarding objectives among varices involved in health care teams [24]. - Clear information and documentation about patient’s history, background, status and prognosis [24]. -Empowered and skilled staff involved in the care process [24]. - Locating physician participation as central during the establishment of comfort care for patients [31]. - Establishing a consensus around decision concerning comfort care [24].
	Withholding or withdrawal of life-sustenance	- Ethical factors influencing doctors’ decision-making processes [33]. - Lack of patients’ advance directives at the time of admission [22, 34, 35, 42]. - Difficulties with palliative care treatment decisions [26].	- Ethics consultations [30].
	Prognostication	- Lack of understanding concerning the assessment of prognostication efforts and pre-death symptoms [24, 25]. - Critical delays in palliative care prognostication and decision-making [28].	- Use numeric prognostic scale [20].
	Multidisciplinary team communication	- Lack of communication and team interaction act as core barriers to providing adequate palliative care in ICU [19, 22]. - Inadequate communication about identification of care objectives between ICU team members and other clinicians [35].	- Multidisciplinary meetings with families to improve communication [22, 34]. - Multidisciplinary team meetings [36].

#### Organizational structures

Several studies highlighted the lack of protocols and policies for integrating a palliative approach in the ICU [22, 23, 34, 37]. Nevertheless, several studies have also revealed that physicians and nurses tend to be resistant to and unaware of the guidelines for a palliative approach in the ICU [26, 37, 38]. The number of staff, the standardization of the staffing ratio [27], and the time spent by staff with patients were examples of factors influencing the integration of a palliative approach in the ICU [19, 34]. There was a lack of organizational support, not in the least for junior nurses, who reported a lack of mentoring and support.

Several studies indicated that poor education and knowledge about a palliative approach created barriers and were due to inadequate education and knowledge among nurses working in the ICU [21, 38]. One study reported that nurses gained their palliative care experience through trial and error [19]. Other barriers identified were insufficient information [32], a lack of awareness of the complexity of and the communication required for a palliative approach in the ICU, inadequate training in palliative care decision making [29, 32], and a lack of specialized palliative care teams, which was an obstacle to integrating a palliative approach for patients in the ICU [37, 40].

A palliative approach was positively enhanced by following standardized tools for dialogue [38], bereavement programmes, and adherence to the appropriate policies and procedures [26]. This approach was also enhanced by the introduction of team meetings [36], collaboration with other specialties [20, 39], the involvement of families into the multidisciplinary discussion [22, 34]. Palliative care training programmes for critical care professionals, or peer-to-peer support programmes, improved the integration of a palliative approach in the ICU [36], as well as specialized palliative care teams’ participation and mentoring within the ICU teams [25].

#### Working environment

The physical and psychosocial care environment was more of a barrier than a facilitator for a palliative approach in the ICU, and only two studies reported that support through a nurse-adapted bedside environment was effective in the ICU [36]. In many studies, the physical environment or infrastructure of the ward did not facilitate the support and participation of families while integrating a palliative approach for their patients [25, 35]. Identified barriers included a noisy environment with lack of privacy and confusion regarding who to approach for information [20, 37, 38]. Increased moral distress and the need for emotional support to reduce such stress during the integration of a palliative approach in the ICU was described in two studies [19, 27], indicating a lack of support from managers, other staff and external support services [27, 32].

#### Patient and family involvement

Three studies emphasized that conflict and disagreements between family members and physicians concerning the goals of care were a barrier to the integration of a palliative approach [22, 32, 35], and other studies highlighted communication challenges with family members [20, 34] related to language and culture [35], religious beliefs [35], and inadequate information about prognosis [31, 41]. Nevertheless, family involvement in patient care regarding the sharing of information, respect for others’ wishes, and cooperation among patients, families and healthcare providers before they decided to change the goals of care towards a palliative orientation was found to be a facilitating factor [28, 36, 38].

#### Palliative care decision-making

Studies report that continued intensive care intervention for patients was a barrier to making decisions about the integration of a palliative approach in the goals of care [31, 41], as well as a lack of understanding about how to assess patients’ prognostication towards palliative care for dying patients and their family [24, 25]. For example, one study found that physicians were unable to identify patients who required a palliative approach to care in the early stage of intensive care [28]. Three studies emphasized that nurses did not contribute to the decision to integrate a palliative approach for their patients in the ICU [28, 29, 42]. Other identified barriers to decision making were physicians’ attitudes and beliefs about palliative care [37, 42], disagreement between physicians [19] and the ICU team regarding the goals of care [27, 36], a lack of standardized care [25], and insufficient communication among team members [19, 22, 35], all of which hampered integration in terms of transitions from life-sustaining interventions to palliative goals (see Table 4)*.*

Clarity, agreement and documentation of the palliative goals of care decisions were identified as facilitators [24], together with ethical consultation [30, 33] and the use of a numeric prognostic scale to support improved prognostication efforts [20].

## Discussion

In this study, we sought to identify barriers to and facilitators of the integration of a palliative approach in intensive care units. To our knowledge, this study is the first systematic review that combines results based on qualitative and quantitative data to illuminate factors influencing a palliative approach in the critical care environment. Our results suggest that the transition from life-sustaining interventions to palliative goals of care in an intensive care context is hindered by both organizational and structural factors (e.g., resources, time constraints, workloads, and work environments) as well as individual factors (e.g., healthcare provider, patient, and family attitudes, communication, interaction and knowledge backgrounds). Our quality assessment suggests that the majority of articles (*n* = 16) were assessed to be of either moderate or moderate-to-high quality. The results from the four articles assessed to have low quality were supported by similar results from other studies included.

Today, a palliative approach to care is characterized by early identification of palliative care needs, adaptation of palliative care knowledge and integration into practice [43]. Given the complex nature of the intensive care context, such knowledge translation may become more challenging. There is no doubt that there is a need for the successful knowledge translation of a palliative approach into the ICU; however, the studies reviewed did not explicitly evaluate this. Through acknowledgment of the complexities involved, we need explicit knowledge translation research demonstrating valid implementation strategies. One way of moving towards knowledge translation is using the PARiHS (Promoting Action on Research Implementation in Health Services) model, which provides important insights for supporting knowledge translation into practice by focusing the implementation process on evidence, context and facilitation. According to the PARiHS model, the context/setting, as well as practice facilitators for change, are as important as the evidence supporting the knowledge. We use this model to discuss the findings from this study.

Organizational structures appeared in this study to be one of the barriers to the achievement of a palliative approach in the ICU. According to the PARiHS model, the context/setting, as well as practice facilitators for change, are as important as the evidence supporting the knowledge. The PARiHS framework demonstrates the need to address leadership and organizational aspects by understanding human relationships [44]. The present study shows that the lack of clinical guidelines and policies for integrating palliative care hinders implementation. Nevertheless, there is also evidence that professionals tend to disregard or be unaware of guidelines for a palliative approach in the ICU, pointing towards the importance of understanding professional perceptions and attitudes towards a palliative approach. This finding is in line with a review by Kahveci [14] that showed the impact of sociocultural factors and the lack of awareness of a palliative approach. Professionals’ perceptions and attitudes, leadership and organizational aspects, as well as patients’ and relatives’ preferences and participation, need to be explored for successful integration. Thus, integration is both an organizational challenge as well as an individual challenge, as the organizational culture is created, sustained or changed by the people who work within the organization [45].

An important aspect of knowledge translation highlighted in the PARiHS framework is the context of care and its environment. Our results found more research emphasizing the challenges that the ICU physical work environment imposes on a palliative approach to care [30, 33, 42]. Interestingly, we found no articles pinpointing facilitating factors for the psychosocial care environment in the ICU. Therefore, future research should further investigate the care environment in various situations, such as how to support staff and reduce the stress of the care environment in general in this setting.

The present study highlights the importance of both patient and family involvement. The importance of family involvement is in line with the findings of previous literature [8, 9]. Ineffective communication, a lack of family education, and a lack of healthcare provider awareness were shown to be key issues underlying conflict between family and physicians. Patient and family involvement are linked to knowledge and education [22, 31], so it is vital to address family education programmes in the ICU. Studies show a palliative approach integrated into reduced patient and family distress [10–13]. This suggests a need for improved palliative care education and training to assess patients’ and families’ needs, wishes, and participation in care and goal setting in terms of a palliative orientation.

In the present study, decision-making to integrate a palliative approach in intensive care is influenced by healthcare professionals’ knowledge and attitudes about the transition from curative-focused to palliative-focused goals of care, which highlights the importance of focusing on healthcare professionals’ goal-setting attitudes and abilities in the integration of a palliative approach [46]. Unsurprisingly, having a clear goal of care on admission to the ICU seems to support professionals in the palliative decision-making process [24]. However, this cannot be considered in isolation, as many complex related factors can affect it, for example, the wishes of the patient and family and difficulties in defining a patient’s prognosis on admission [35]. Studies regarding nurses’ involvement in team decision-making or consulting the specialized palliative care team were scarce for palliative care in the ICUs [28, 29, 42]. Further studies may explore the impact of nurses’ involvement in the decision-making process.

### Limitations of the study

In this systematic literature review, we described factors influencing the integration of a palliative approach within the ICU. The majority of the included studies were assessed to be of moderate or moderate-to-high quality, with only one of these assessed to be of high quality, and four studies were assessed to be of low quality. The reader should thus acknowledge the heterogeneity of the study designs, as well as the spectrum of quality within the included studies. Although the heterogeneity of studies within a mixed-methods review could be acknowledged as a limitation, it is also a strength, as it provides a broad overview of the topic. As in many systematic studies, researchers’ language skills are a limitation because we only included literature in English.

## Conclusion

Factors hindering the integration of a palliative approach in an intensive care context are constituting a complex interplay among the organizational structure, the care environment and the clinician’s perception and attitudes. While patient and family involvement were identified as an important facilitator of palliative care, it was also identified as a barrier for the clinicians due to challenges in shared goal setting and communication. We suggest that future integration efforts targeting a palliative approach should focus on organizational and educational efforts that strengthen human relationships and partnerships, not in the least regarding patient and family involvement. Moreover, there is a need for research evaluating useful strategies for the knowledge translation of a palliative approach in the ICU.

## Supplementary information

**Additional file 1.** All databases searched form 2017-12-22 to 2018-01-31.

**Additional file 2.** Quality assessment A1–A4.

## Data Availability

Data sharing is not applicable to this article, as no datasets were generated or analysed during the current study.

## Abbreviations

ICU: Intensive care units
RCT: Randomized control trial
CASP: Critical Assessment Skills Program
MMAT: The Mixed Methods Appraisal Tool
Best BETs: Best Evidence topics
PARiHS: Promoting Action on Research Implementation in Health Services
PICOC: Population, Intervention, Comparison, Outcome and Context
