# Supplementary material for: Factors influencing the integration of a palliative approach in intensive care units: a systematic mixed-methods review
Source: BMC Palliat Care. 2020 Jul 22;19:113. doi: 10.1186/s12904-020-00616-y (PMC7375204; doi:10.1186/s12904-020-00616-y)
Supplement: Supplementary file 1 — Additional file 1. All databases searched form 2017-12-22 to 2018-01-31. [file 12904_2020_616_MOESM1_ESM.docx]

# **The data bases search**

# **All databases searched form 2017-12-22 to 2018-01-31**

| **Databases** | **Search term** |
| --- | --- |
| AMED | **Implementation**  exp Health Services Research/ OR Regional Health Planning OR critical pathways.mp. OR advance care planning.mp. OR exp Guidelines/ OR exp Delivery of Health Care/ OR capacity building .mp. OR exp Education Professional/ OR inservice training.mp. OR exp Ethics Professional/ OR Medical Audit OR Program Development OR translational research.mp. OR translational medicine.mp. OR translational medical research.mp. OR Evidence based Medicine OR (Implementation science or implementation research or knowledge dissemination or diffusion of innovation or knowledge translation or knowledge transfer or knowledge exchange or improvement science).ti. OR (Implementation science or implementation research or knowledge dissemination or diffusion of innovation or knowledge translation or knowledge transfer or knowledge exchange or improvement science).ab. OR (Implementation science or implementation research or knowledge dissemination or diffusion of innovation or knowledge translation or knowledge transfer or knowledge exchange or improvement science)  AND  **Palliative care**  (Terminally ill or Terminal illness or Terminal care or end of life or palliative treatment or palliative medicine or hospice care or hospices).ti. OR (Terminally ill or Terminal illness or Terminal care or end of life or palliative treatment or palliative medicine or hospice care or hospices).ab. OR (Terminally ill or Terminal illness or Terminal care or end of life or palliative treatment or palliative medicine or hospice care or hospices).hw. OR palliative care/ OR terminal care/ OR hospice care/  AND  **Intensive care units**  exp critical care/ OR exp intensive care |
| Result | Total 🡪 21 |
| Limited | Limits 2007-2018 and English 🡪 21 |
| Endnote | Endnote export 🡪 21 2018-01-31 |

| **Databases** | **Search term** |
| --- | --- |
| PsycInfo | **Implementation**  ab("Evidence Based Medicine" OR "Health Plan Implementation" OR "Regional Health Planning" OR "Advance Care Planning" OR "Critical Pathways" OR "Guideline Adherence" OR ("Delivery of health care" AND integrated) OR "Capacity Building" OR "Professional Education" OR "Ethics Consultation" OR "Clinical Audit" OR "Translational Medical Research" OR "Translational Research" OR "Translational Medicine" OR "Implementation science OR implementation research OR knowledge dissemination OR diffusion of innovation" OR "knowledge translation" OR "knowledge transfer" OR "knowledge exchange" OR "improvement science") OR ti("Evidence Based Medicine" OR "Health Plan Implementation" OR "Regional Health Planning" OR "Advance Care Planning" OR "Critical Pathways" OR "Guideline Adherence" OR ("Delivery of health care" AND integrated) OR "Capacity Building" OR "Professional Education" OR "Ethics Consultation" OR "Clinical Audit" OR "Translational Medical Research" OR "Translational Research" OR "Translational Medicine" OR "Implementation science OR implementation research OR knowledge dissemination OR diffusion of innovation" OR "knowledge translation" OR "knowledge transfer" OR "knowledge exchange" OR "improvement science") OR (MAINSUBJECT.EXACT.EXPLODE("Organizational Climate") OR MAINSUBJECT.EXACT.EXPLODE("Treatment Guidelines") OR MAINSUBJECT.EXACT.EXPLODE("Continuing Education") OR MAINSUBJECT.EXACT.EXPLODE("Inservice Training") OR MAINSUBJECT.EXACT.EXPLODE("Program Development") OR MAINSUBJECT.EXACT.EXPLODE("Evidence Based Practice") OR MAINSUBJECT.EXACT.EXPLODE("Best Practices") OR MAINSUBJECT.EXACT.EXPLODE("Clinical Audits")) 🡪 57040  AND  **Palliative care**  (MAINSUBJECT.EXACT.EXPLODE("Palliative Care") OR MAINSUBJECT.EXACT.EXPLODE("Hospice") OR MAINSUBJECT.EXACT.EXPLODE("Terminally Ill Patients") OR MAINSUBJECT.EXACT.EXPLODE("Treatment Withholding")) OR (AB("Palliative Care" OR hospice* OR "hospice care" OR "Terminally Ill Patients" OR "terminally ill" OR "End of life" OR "end-of-life" OR "end-of-life care" OR "Terminal Care" OR "palliative treatment" OR "palliative medicine" OR "comfort care" OR "supportive care" OR "withholding treatment" ) OR TI("Palliative Care" OR hospice* OR "hospice care" OR "Terminally Ill Patients" OR "terminally ill" OR "End of life" OR "end-of-life" OR "end-of-life care" OR "Terminal Care" OR "palliative treatment" OR "palliative medicine" OR "comfort care" OR "supportive care" OR "withholding treatment" )) 🡪 37471  AND  **Intensive care units**  MAINSUBJECT.EXACT.EXPLODE("Intensive Care") OR "critical care" OR ICU |
| Result | Total 🡪 64 |
| Limited | Limit Time 2007-01-01 – 2018-01-01 🡪 53 träffar  Limit Peer reviewed 🡪 47 Limit languages (Eng., German) 🡪 47 |
| Endnote | Endnote export 🡪 46 |

| **Databases** | **Search term** |
| --- | --- |
| Sociological Abstracts | **Implementation**  ti((("Evidence Based Medicine" OR "Health Plan Implementation" OR "Regional Health Planning" OR "Advance Care Planning" OR "Critical Pathways" OR "Guideline Adherence" OR ("Delivery of health care" AND integrated) OR "Capacity Building" OR "Professional Education" OR "Ethics Consultation" OR "Clinical Audit" OR "Translational Medical Research" OR "Translational Research" OR "Translational Medicine" OR "Implementation science" OR "implementation research" OR "knowledge dissemination" OR "diffusion of innovation" OR "knowledge translation" OR "knowledge transfer" OR "knowledge exchange" OR "improvement science"))) OR ab((("Evidence Based Medicine" OR "Health Plan Implementation" OR "Regional Health Planning" OR "Advance Care Planning" OR "Critical Pathways" OR "Guideline Adherence" OR ("Delivery of health care" AND integrated) OR "Capacity Building" OR "Professional Education" OR "Ethics Consultation" OR "Clinical Audit" OR "Translational Medical Research" OR "Translational Research" OR "Translational Medicine" OR "Implementation science" OR "implementation research" OR "knowledge dissemination" OR "diffusion of innovation" OR "knowledge translation" OR "knowledge transfer" OR "knowledge exchange" OR "improvement science"))) OR (MAINSUBJECT.EXACT.EXPLODE("Evidence Based Practice") OR MAINSUBJECT.EXACT.EXPLODE("Implementation") OR MAINSUBJECT.EXACT.EXPLODE("Health Planning") OR MAINSUBJECT.EXACT.EXPLODE("Professional Training") OR MAINSUBJECT.EXACT.EXPLODE("Job Training") OR MAINSUBJECT.EXACT.EXPLODE("Work Environment") OR MAINSUBJECT.EXACT.EXPLODE("Organizational Culture")))  AND  **Palliative care**  ((MAINSUBJECT.EXACT.EXPLODE("Hospices") OR MAINSUBJECT.EXACT.EXPLODE("Terminal Illness") OR MAINSUBJECT.EXACT.EXPLODE("Palliative Care")) OR ab(("Palliative Care" OR hospice* OR "hospice care" OR "Terminally Ill Patients" OR "terminally ill" OR "End of life" OR "end-of-life" OR "end-of-life care" OR "Terminal Care" OR "palliative treatment" OR "palliative medicine" OR "comfort care" OR "supportive care" OR "withholding treatment")) OR ti(("Palliative Care" OR hospice* OR "hospice care" OR "Terminally Ill Patients" OR "terminally ill" OR "End of life" OR "end-of-life" OR "end-of-life care" OR "Terminal Care" OR "palliative treatment" OR "palliative medicine" OR "comfort care" OR "supportive care" OR "withholding treatment")))  AND **Intensive care units**  ("intensive care" OR "critical care" OR ICU) |
| Result | Total 🡪 5 |
| Limited | Limit 2007-01-01-2018-01-01 🡪 4  Limit Peer reviewed 🡪 4 Limit languages (Eng) 🡪 3 |
| Endnote | Endnote export 🡪 3 |

| **Databases** | **Search term** |
| --- | --- |
| Embase | **Implementation**  'health care planning'/exp OR 'protocol compliance'/exp OR 'integrated health care system'/exp OR 'capacity building'/exp OR 'in service training'/exp OR 'continuing education'/exp OR 'vocational education'/exp OR 'clinical audit'/exp OR 'translational research'/exp OR 'evidence based nursing'/exp OR 'evidence based nursing'/de OR 'ethics consultation':ti,ab,kw OR 'implementation science':ti,ab,kw OR 'implementation research':ti,ab,kw OR 'knowledge dissemination':ti,ab,kw OR 'diffusion of innovation':ti,ab,kw OR 'knowledge translation':ti,ab,kw OR 'knowledge transfer':ti,ab,kw OR 'knowledge exchange':ti,ab,kw OR 'improvement science':ti,ab,kw  AND  **Palliative care**  'palliative therapy'/exp OR 'terminal care'/de OR 'terminally ill patient'/exp OR 'hospice care'/exp OR 'hospice'/exp OR 'advance care planning'/exp OR 'palliative nursing'/exp OR 'treatment withdrawal'/de OR 'supportive care'/exp  AND  **Intensive care units**  ‘Intensive Care’/exp OR ‘Intensive Care Unit’/exp |
| Result | Total 🡪 486 |
| Limited | Limit 2007 -2018 🡪 376 |
| Endnote | Endnote export 🡪 376 |

| **Databases** | **Search term** |
| --- | --- |
| Web of Science | **Implementation** (TS=("Health Plan Implementation" OR "Regional Health Planning" OR "Advance Care Planning" OR "Critical Pathways" OR "Guideline Adherence" OR "Practice Guidelines as topic" OR "Delivery of Health Care, Integrated" OR "Capacity Building" OR "Education, Professional" OR "Inservice Training" OR "Ethics Consultation" OR "Clinical Audit" OR "Program Development" OR "Translational Medical Research" OR "Evidence-Based Practice" OR "Organizational Culture" OR “Implementation science” OR “implementation research” OR “knowledge dissemination” OR “diffusion of innovation” OR “knowledge translation” OR “knowledge transfer” OR “knowledge exchange” OR “improvement science”  AND  **Palliative care**  TS=(palliative* OR terminal* OR hospice* OR "end of life")  Indexes=SCI-EXPANDED, SSCI, A&HCI, CPCI-S, CPCI-SSH, ESCI  AND  **Intensive care units**  TS=("intensive care" OR "intensive unit" OR ICU OR "critical care") |
| Result | Total 🡪 200 |
| Limited | Limit 2007 -2018 🡪 165 Limit languages (Eng) 🡪160 |
| Endnote | Endnote export 🡪 160 |

| **Databases** | **Search term** |
| --- | --- |
| Cinahl | **Implementation**  MH ( (Health and Welfare Planning OR State Health Plans OR Patient Care Plans OR Nursing Care Plans OR Health Facility Planning OR Medical Practice, Research-Based, OR Nursing Practice, Research-Based OR Professional Practice, Evidence-Based OR System implementation OR Program Development OR Advance Care Planning OR Critical Path OR Guideline Adherence OR Practice guidelines OR Health Care Delivery, Integrated OR Education, Continuing OR Staff Development OR Ethics Committees OR Staff development OR Organizational culture OR Quality Improvement OR Diffusion of Innovation) ) OR ( TI Implementation science OR AB Implementation science OR TI implementation research OR AB implementation research OR TI knowledge dissemination OR AB knowledge dissemination OR TI knowledge translation OR AB knowledge translation OR TI knowledge transfer OR AB knowledge transfer OR TI knowledge exchange OR AB knowledge exchange OR TI improvement science OR AB improvement science OR TI translational medical research OR AB translational medical research )  AND  **Palliative care**  MH (Palliative Care OR (Hospice and Palliative Nursing) OR Hospices OR Hospice Patients OR Hospice Care OR Terminal Care OR Terminally Ill Patients OR End of life)  AND  **Intensive care units**  critical care OR intensive care OR (MH "Intensive Care Units") |
| Result | Total 🡪 332 |
| Limited | Limit Peer reviewed 🡪312 Limit 2007-2018 🡪 240 Limit languages (Eng) 🡪 235 |
| Endnote | Endnote export 🡪 235 |

| **Databases** | **Search term** |
| --- | --- |
| Scopus | **Implementation**  TITLE-ABS-KEY =("Health Plan Implementation" OR "Regional Health Planning" OR "Advance Care Planning" OR "Critical Pathways" OR "Guideline Adherence" OR "Practice Guidelines as topic" OR "Delivery of Health Care, Integrated" OR "Capacity Building" OR "Education, Professional" OR "Inservice Training" OR "Ethics Consultation" OR "Clinical Audit" OR "Program Development" OR "Translational Medical Research" OR "Evidence-Based Practice" OR "Organizational Culture" OR “Implementation science” OR “implementation research” OR “knowledge dissemination” OR “diffusion of innovation” OR “knowledge translation” OR “knowledge transfer” OR “knowledge exchange” OR “improvement science”)  AND  **Palliative care**  TITLE-ABS-KEY ((palliative* OR terminal* OR hospice* OR "end of life" OR dying )  AND  **Intensive care units**  ( TITLE-ABS-KEY ( "intensive care" OR "intensive unit" OR icu OR "critical care" ) ) |
| Result | Total 🡪 614 |
| Limited | Limit 2007-2018 🡪 513 Limit languages (Eng) 🡪 495 |
| Endnote | Endnote export 🡪 495 |

| **Databases** | **Search term** |
| --- | --- |
| PubMed | **Implementation**  Health Plan Implementation[MH] OR Regional Health Planning[MH] OR Advance Care Planning[MH] OR Critical Pathways[MH] OR Guideline Adherence[MH] OR Practice Guidelines as topic[MH] OR Delivery of Health Care, Integrated[MH] OR Capacity Building[MH] OR Education, Professional[MH] OR Inservice Training OR Ethics Consultation[MH] OR Clinical Audit[MH] OR Program Development[MH] OR Translational Medical Research[MH] OR Evidence-Based Practice[MH] OR Organizational Culture[MH] OR Implementation science [tiab] OR implementation research [tiab] OR knowledge dissemination [tiab] OR diffusion of innovation [tiab] OR knowledge translation [tiab] OR knowledge transfer [tiab] OR knowledge exchange [tiab] OR improvement science [tiab]  AND  **Palliative care**  Palliative Care[Mesh] OR Palliative[tiab] OR Hospice[tiab] OR Hospices[tiab] OR Hospice and Palliative Care Nursing[Mesh] OR Palliative Medicine[Mesh] OR Terminal Care[Mesh] OR Hospice Care[Mesh] OR Hospices[Mesh] OR Hospice[tiab] OR Hospices[tiab] OR "End of life"[tiab] OR "End-of-life"[tiab] OR "withholding treatment"[tiab] OR "supportive care"[tiab] OR "comfort care"[tiab]  AND  **Intensive care units**  Intensive care units[mesh] OR intensive care[mesh] OR “intensive care units”[tiab] OR” intensive care”[tiab] OR Critical Care[mesh] OR “Critical Care”[tiab] OR ”intensive unit”[tiab] OR “intensive therapy unit”[tiab] OR “intensive treatment unit”[tiab] OR “critical care unit”[tiab] OR ICU[tiab] OR CCU[tiab] OR ITU[tiab] |
| Result | Total 🡪 798 |
| Limited | Limit 2007-2018 🡪 528 Limit languages (Eng) 🡪 507 |
| Endnote | Endnote export 🡪 507 |
