# Supplementary material for: Factors influencing the integration of a palliative approach in intensive care units: a systematic mixed-methods review
Source: BMC Palliat Care. 2020 Jul 22;19:113. doi: 10.1186/s12904-020-00616-y (PMC7375204; doi:10.1186/s12904-020-00616-y)
Supplement: Supplementary file 2 — Additional file 2. Quality assessment A1–A4. [file 12904_2020_616_MOESM2_ESM.docx]

**Additional file 2: Quality assessment A1–A4**

**Critical appraisal skill programme (CASP)**

- **A1:** Quality assessment of the nine qualitative articles

**Rating scale: (1–2 Low) (3–4 Moderate low) (5–6 Moderate) (7–8 Moderate high) (9–10 High)**

| CASP Quality assessment (A qualitive study). Rating scale (1–2 Low) (3–4 Moderate low) (5–6 Moderate) (7–8 Moderate high) (9–10 High) | | | | | | | | | | |
| --- | --- | --- | --- | --- | --- | --- | --- | --- | --- | --- |
| CASP | **1.Was there a clear**  **statement of the aims of**  **the research?** | **2. Is a qualitative methodology appropriate?** | **3. Was the research**  **design appropriate to**  **address the aims of the**  **research?** | **4. Was the recruitment**  **strategy appropriate to**  **the aims of the**  **research?** | **5. Was the data collected in**  **a way that addressed the**  **research issue?** | **6. Has the relationship between researcher and participants been adequately considered?** | **7. Have ethical issues been taken into consideration?** | **8. Was the data analysis sufficiently rigorous?** | **9. Is there a clear statement of findings?** | **10. How valuable is the research?** |
| (26) Zomorodi  2010 (USA)  Title:  Critical Care Nurses’ Values and Behaviours With End-of-Life Care: Perceptions and Challenges  **(7–8 Moderate high)** | **Yes,** the aim of this study is clear:  To explore nurses’ definitions of quality EOL care and to describe the activities that promote quality EOL care in the ICU.  It is a relevant and important aim because an individual should recognize their values and how these values affect their behaviour. | **Yes,** the study seeks to understand and describe the definition of quality EOL care and this method is appropriate for this type of question. The researcher did not emphasize why he/she used this type of method in the paper. | **No,** the researcher did not explain how he/she decided which method to use in this study. | **Yes;** however, some of the  limitation  covers the recruitment.  The researcher explained how the participants were recruited; however, the researcher did not state the reasons for choosing the participants they selected. | **Yes; s**ome information about the setting (adult ICUs) is mentioned.  The data were collected via interview  (semi-structured) and the researcher chose this method due to the emotional and sensitive nature of palliative care.  The researcher indicated some specific details of how interviews were conducted.  For example, the participants were asked to respond to the following questions, etc.  The interviews were audiotaped.  Field notes taken after each interview were used by the researcher.  The researcher considered the saturation of data. | **No** existing relationship is documented in this research paper. | **Yes**, ethical approval was considered; however, it is not adequately described by the researcher.  For example,  confidentiality and handling of the data should be detailed, as well as how the researcher defined and maintained the ethical standards for the participants. | **Yes,** there is brief description of the analysis process.  Thematic analysis was used, but it is not clear how the categories/themes were derived from the data. However, the researcher provides quotes from the interviews.  The researcher describes how they had the data analysed by an expert researcher to mitigate the risk of bias.  The data were sufficient to support the findings. | **Yes,** The findings of this study are clear and described in sufficient detail.  The findings of this study were discussed in relation to  the research aim. | As I read this study, I felt this is a valuable outcome and article.  The aim of the study was answered.  The author discussed their findings in relation to current  practice and policy;  for example, the curative model of care.  The researcher identified new areas where further research  is warranted.  The paper included discussion on  how the results can be transferred to  other  populations, with suggestions. |
|  |  |  |  |  |  |  |  |  |  |  |
| (25) Holms.et al,  2014 (UK)  Title:  A study of the lived experiences of registered nurses who have provided end-of-life care within an intensive care unit  **(3–4 Moderate low)** | **Yes,** the aim of this study is clear:  To explore the experiences of ICU nurses who have provided EOL care to patients and their families. | **Yes,** the researcher seeks to explore participants’ live experiences by phenomenological design, which is appropriate. | **Yes,** a clear statement is made about why a qualitative method is appropriate for this study.  (Methods section) 550 | **Yes**, the researcher has explained how and why the  participants were selected. For example, in this study the participants were selected according to clear inclusion criteria, and this was  appropriate to provide access to the  type of knowledge  required by the study.  The researcher discussed during the  recruitment process that nurses who are distressed or uncomfortable talking about EOL care do not have to take part. However, some of the  limitation  covers the recruitment. | **Yes,** the data collection was held in a comfortable room in the general ICU.  The data were collected via interview  (semi-structured, open-ended questions).  The researcher indicated some details of how interviews were conducted:  comfortable room, time of interview, audiotaped. | **Yes,** there appears to be a relationship because the researcher states that participants are from the workplace (recruitment section).  However, the author mentions that this relationship could influence the result. | **Yes,** there are some details of ethical process such as how the participants are enrolled in this study, via an invitation letter and information sheet. However, there is no detail regarding data confidentiality or board ethical approval. | **Yes,** the researcher has transcribed and written an in-depth description of the analysis process. The researcher used thematic analysis.  The researcher mentions the number of categories and themes, but it is not clear how the categories/themes were derived from the data.  There are some examples that were derived from original data (showing the analysis process).  The findings are sufficient and support the aim of the study.  The researcher analysis and selection of the data involved peer review to reduce potential bias. | \| **Yes,** The findings of the study are clear.  The research discussion is also clear and adequate.  The findings were discussed in relation to the research question. \| \| --- \| | The study is valuable, from what I read of the results and discussion parts; however, there is no new result from this study according to the author’s perception.  The researchers discussed the limitations of the study. |
| (23) Walker, R,  2010  (UK)  Title: The Liverpool Care Pathway in intensive care: an exploratory study of doctor and nurse perceptions  **(5–6 Moderate)** | **Yes**, the aim of this study is clear:  To explore doctor and nurse experiences of the impact of the LCP in two intensive care units. | **Yes,** the researcher seeks to interpret and explore participant experiences. I believe qualitative research is the right  methodology for this  research aim. | **Yes,** a clear statement is made about why a qualitative method is appropriate for this study.  (Methods section) | **Yes,** the researcher explains how the participants were chosen according to an inclusion strategy. However, there is no inclusion strategy mentioned in the study, other than nurses and doctors who have used LCP.  The researcher stated that variation of the experience could help in finding common themes.    There was no discussion on why other participants did not take part in the study. | **Yes,** data collection was addressed: semi-structured interviews were held with ICU staff using a digital recorder.  Guided questions were also used in the study conducted in the ICU at one hospital. | **No** existing relationship is documented in the paper.  There were no potential biases regarding the formulated research questions or data collection, for example recruitment, but the researcher stated that she is a nurse and had no experience in ICUs. | **Yes, can’t tell** Ethical approval was covered by a subtitle in this study, and the study was approved by committee. However, information is lacking about how the research was explained and how the participants were enrolled ethically in this study (for example, a participant information form).  There also is not enough information about ethical issues around  informed consent, confidentiality, etc. | **Yes,** there is clear description of the analysis process.  Simultaneous thematic analysis was used.  It is clear  how the categories/themes were derived  from the data.  The researcher explains how the  data were obtained, and how the data were selected to demonstrate the analysis process.  The data are presented and support the findings.  The researcher analysed the data independently with another researcher. There was no critical examination of potential bias during analysis. | **Yes,** the findings showed clear themes that were mentioned in one part and were described very well and explicitly (269).  There is adequate discussion of the  evidence.  The researcher has discussed the  credibility of the findings. The results are also discussed in relation to  the aim of the study. | **Yes,** I think the study is valuable. The researcher covers new areas where research is necessary and  considers the findings in relation to current practice or policy, or relevant research-based literature. |
| (33) Anderson  2015  (USA)  Title: A multicentre study of key stakeholders' perspectives on communicating with surrogates about prognosis in intensive care units  **(7–8 Moderate high)** | **Yes,** the aim of this study is clear:  To determine the perspectives of key stakeholders regarding how prognostic information should be conveyed in critical illness. | **Yes,** the researcher seeks to describe the perception of participants on how the prognosis should be communicated. I believe qualitative research is the right  methodology for this  research aim. | **No ,** there is no clear statement about why a qualitative method is appropriate for this study. | **Yes,** the researcher explains how the three types of participant were recruited and an exclusion strategy is mentioned. the researcher uses a purposeful sample. | **Yes,** the data were collected via interview  (semi-structured) and the researcher has chosen this method  to elicit information from participants on their perception of how prognoses should be communicated.  The researcher indicated some details of how interviews were conducted: ICU setting, conference room, interview time, digital recorder. | No existing relationship is documented in this research paper. | **Yes**, ethical approval was considered and obtained. Examples of informed and verbal consent are given.  There is not enough description of, for example,  confidentiality and handling of the data, etc., or of how the researcher defined and maintained ethical standards for the participants. | **Yes,** there is clear description of analysis of the interviews.  Thematic analysis was used.  It is clear  how the themes were derived  from the data (Tables).  The researcher explains how the  data were obtained and how the data were examined for thematic saturation.  The data are presented in text and tables. The data in this study support the findings.  The researcher analysed the data independently with other researchers. There was critical examination of the potential bias during analysis. | **Yes,** the findings showed clear themes that were mentioned in one part and were described very well and explicitly.  There is adequate discussion of the  evidence.  The researcher has discussed the  credibility of findings. The results are discussed in relation to  the aim of study. | **Yes,** the researcher stated how the findings can be transferred. The researcher recommended using the participants’ perceptions in practical interventions and guidelines. |
| (20) Baggs et al, 2007  (USA)  Title: Intensive care unit culture and end-of-life decision making  **(3–4 Moderate low)** | **Yes,** the aim of this study is clear:  To study limitations of treatment decision making in real time, and to evaluate similarities and differences in the cultural contexts of four ICUs and the relationship of those contexts to EOL decision making. | **Yes,** the researcher seeks to understand the similarities and differences in the cultural contexts of four ICUs and the relationship of those contexts to EOL decision making.  I believe qualitative research is the right  methodology for this  research aim. | **Yes,** there is a clear statement about why an ethnographic method is appropriate for this study.  (Methods section) | **Can’t tell –** the researcher does not explain how the participants were chosen.  In addition, no inclusion strategy was mentioned in the study.  The researcher mentioned when the data collection was started.  There was no discussion on why other participants did not take part in the study. | **Yes,** the data were collected via interview  (semi-structured, open-ended questions).    The researcher has also used interview guide field notes.  The researcher has chosen this collection method because it is based on perception of decision making, barriers and facilities.    The researcher indicated some details of how interviews were conducted:  5 hours a day every week over 7 months.  The researcher has not discussed saturation of data. | **No** existing relationship is documented in this research paper. | **Yes**, ethical approval was considered and obtained. Examples: informed consent, information sheet.  There is adequate description of confidentiality and handling of the data, etc.  There is insufficient information on how the researcher defined and maintained ethical standards for the participants. | **Yes,** there is clear description of research analysis  in pp. 161–162.  Thematic analysis was used.  It is clear  how the themes were derived  from the data;  some examples  are included.  There is inadequate explanation of how the data were thematically saturated.  The data are presented in the text. The data in this study support the findings.  The researcher analysed the data with a research team. | **Yes, t**he findings of the study are clearly described.  There is adequate discussion of the results.  The findings were discussed in relation to the research question.  The credibility of the findings was discussed. | **Yes,**  I think the study is valuable, and the findings identify new areas of research (attention to structure, culture and variation in providing roles for specific units).  However, the researcher did not state how the findings can be transferred to other populations or settings. |
| (21) Ranse, K,  2012  (Australia)  Title: End-of-life care in the intensive care setting: A descriptive exploratory qualitative study of nurses’ beliefs and practices  **(7–8 Moderate high)** | **Yes,** the aim of this study is clear: To explore the end-of-life care beliefs and practices of intensive care nurses. | **Yes,** the researcher seeks to understand and explore the  end-of-life care beliefs and practice of intensive care nurses.  Descriptive exploratory qualitative study involves understanding and summarizing, and I think this method is the right methodology for this research aim. | **Yes,** there is a clear statement about why descriptive exploratory qualitative study is appropriate for this study.  (Methods section) 6 | **Yes,** the researcher explained how the participants were chosen.  Registered nurses (senior and junior staff) were eligible.  There is no inclusion strategy mentioned in the study.  The researcher stated how the participants were recruited.  There was no discussion on why other participants did not take part in the study. | **Yes,** the data were collected via interview questions  (semi-structured, open-ended questions).    The researcher has justified the methods chosen: this provides participants with the opportunity to openly share personal experiences of providing end-of-life care.  The researcher indicated some details of how interviews were conducted.  The researcher has not discussed saturation of data. | **No** existing relationship is documented in this research paper. | **Yes**,  ethical approval was considered.  There is inadequate description of confidentiality and handling of the data, etc.  There is inadequate explanation of how the researcher defined and maintained ethical standards for the participants. | **Yes,** there is clear description of the research analysis.  An inductive code approach was used to develop categories.  It is clear  how the categories  were derived  from the data;  some examples  are included (text and tables).  There is insufficient explanation of how the  data were examined for category saturation.  The data are presented in the text. The data in this study support the findings.  The researcher  did not examine the role of potential bias and influence during analysis and selection of data. | **Yes,** the findings are clear, and categories  were mentioned in one part and were described very well and explicitly.  There is adequate discussion of the  findings.  The researcher has applied some analysis of the  credibility of findings.  The results are discussed in relation to  the aim of the study. | **Yes,** I think the study is valuable.  The researcher considered  the findings in relation to current practice and relevant evidence-based literature.  The researcher identified new areas where research is necessary (family experience of being involved with end-of-life care).  The researcher has not discussed whether the findings can be transferred to other populations or settings. |
| (39) Radcliffe, C,  2015  (UK)  Title: Use of a supportive care pathway for end-of-life care in an intensive care unit: a qualitative study  **(5–6 Moderate)** | **Yes,** the aim of this study is clear:  To assess the views and experiences of clinical nurses using a supportive care pathway. | **Yes,** the researcher seeks to understand the perception of palliative care held by healthcare professionals in  intensive care. | **Yes,** there is a clear statement about why qualitative study is appropriate for obtaining the views and experiences of nursing staff . | **Yes,** the researcher explained how the participants were chosen.  The researcher explained why the participants they selected were chosen.  The sample consisted of registered nurses and physicians working with an SCP.  There is a clear inclusion strategy mentioned in the study.  The researcher stated how the participants were recruited.  There was discussion on why other participants did not take part in the study. | **Yes,** the data were collected via interview questions  (semi-structured, open-ended questions).    The researcher has justified the methods chosen.  There is an indication of how interviews were conducted, using guiding questions and face-to-face interviews.  The researcher has given a clear description of how interviews were conducted.  The researcher has discussed saturation of data. | **No** existing relationship is documented in this research paper. | **Yes**,  ethical approval was obtained.  There is adequate explanation of how the researcher defined and maintained ethical standards for the participants, e.g. a consent form.  There is insufficient description of confidentiality and handling of the data, etc. | **Yes,** there is clear description of the research analysis.  Thematic analysis was used.  It is clear  how the themes were derived  from the data;  some examples  are included.  The researcher gives adequate explanation of how the  data were examined for thematic saturation.  The data are presented in the text. The data in this study support the findings.  The researcher analysed the data with a second researcher.  There is adequate explanation of how the  data were examined for category saturation.  The researcher  did not examine the role of potential bias and influence during analysis and selection of data. | **Yes,** the findings are explicit and themes  were mention  there is adequate discussion of the evidence  the findings are discussed in relation to the original research question  the researcher has discussed the credibility of findings | **Yes,** I think the study is valuable  The researcher consider the findings in relation to current practice  identify new areas where research is necessary)  The researcher stated that the findings limitation for transferred to other populations |
| (34) Gulini,  2017  (Brazil)  Title: Intensive Care Unit team perception of palliative care: the discourse of the collective subject  **(7–8 Moderate high)** | **Yes,** the aim of this study is clear.  This study describes the views of health professionals using a supportive care pathway in intensive care. | **Yes,** the researcher seeks to understand the perception of palliative care held by healthcare professionals in  intensive care. | **Yes,** there is a clear statement about why a qualitative method is appropriate for this study. | **Yes,** the researcher explained how the participants were chosen.  The researcher explained why the participants they selected were chosen (2 method section).  The sample consisted of registered nurses, nursing technicians, physicians and physical therapists.  There is a clear inclusion strategy mentioned in the study.  The researcher stated how the participants were recruited.  There was discussion on why other participants did not take part in the study. | **Yes,** the data were collected via interview questions  (semi-structured, open-ended questions).    The researcher has justified the methods chosen.  There is an indication of how interviews are conducted, using two guiding questions, and an individual recorder.  The researcher gave a clear description of how interviews were conducted.  The researcher has discussed saturation of data. | **No** existing relationship is documented in this research paper. | **Yes**, ethical approval was obtained.  There is inadequate description of confidentiality and handling of the data, etc.  There is adequate explanation of how the researcher defined and maintained ethical standards for the participants. | **Yes,** there is clear description of research analysis within four steps, as well as use of software (2).  There is discourse on the collective subjective and central idea.  It is fairly clear  how the categories  were derived  from the data;  some examples  are included (text).  There is inadequate explanation of how the  data were examined for category saturation.  The data in this study support the findings.  The researcher  did not examine the role of potential bias and influence during analysis and selection of data. | **Yes,** the findings are explicit and categories  were mentioned as DCS and CI and were described well.  There is adequate discussion of the evidence.  The findings are discussed in relation to the original research question.  The researcher has not discussed the credibility of the findings. | **Yes,** I think the study is valuable.  The researcher considers the findings in relation to current practice.  The need for building care protocol for PC patients is discussed (identifying new areas where research is necessary).  The researcher stated that the findings had limitations in transferability to other populations. |
| (27) Liaschenko, J, 2009  (USA)  Title: The ‘‘Big Picture’’  Communicating With Families About End-of-Life Care in Intensive Care Unit  **(5–6 Moderate)** | **Yes,** the aim of this study is clear.  This study investigates factors influencing critical care nurses’ provision of end-of-life care and their inclusion of families in that care. | **Yes,** the researcher seeks to understand the factors influencing critical care nurses’ provision of end-of-life care. | **No,** the researcher has not clearly justified how he/she decided which method to use. | **Yes,** the researcher explained how the participants were chosen.  The researcher explained why the participants they selected were chosen (method section).  The sample consisted of critical care registered nurses, who worked in medical surgical cardiac ICUs.  There is a clear inclusion strategy mentioned in the study.  The researcher stated how the participants were recruited. | **Yes,** the data were collected via a focus group with an interview guide.  The researcher has not justified the methods chosen.  There is an indication of how focus groups were conducted, using four guiding questions, focus group sessions, and an audiotape.  The researcher gave a clear description of how interviews were conducted.  The researcher has not discussed or considered saturation of data. | **No** existing relationship is documented in this research paper. | **Yes**,  ethical approval was obtained.  There is inadequate description of confidentiality and handling of the data, etc.  There is adequate description of how the researcher defined and maintained ethical standards for the participants. | **Yes,** there is clear in-depth description of the research analysis within several steps.  The researcher analysed the data using content analysis.  There is fairly clear description of  how the final themes were derived  from the data;  some examples  are included (text).  there is inadequate explanation of how the  data were coded, categorized, and included in the process of saturation.  The data in this study support the findings.  The researcher  examined the role of potential bias and influence during analysis and selection of data. | **Yes,** the findings are explicit and themes  were mentioned.  There is adequate discussion of the evidence.  The findings are discussed in relation to the original research question.  The researcher has discussed the credibility of the findings. | **Yes,** I think the study is valuable.  The researcher has considered the findings in relation to current practice.  The need for building care following strategies for families of patient end-of-life care is discussed (identifying new areas where research is necessary).  The researcher stated that the findings had limitations in transferability to other populations. |

**Critical appraisal skill programme (CASP)**

- **A2:** Quality assessment of RCT

**Rating scale: (1–2 Low) (3–4 Moderate low) (5–6 Moderate) (7–8 Moderate high) (9–10 High)**

|  | **1. Did the trial address a clearly focused issue?** | **2. Was the assignment of patients to treatments randomized?** | **3. Were all of the patients who entered the trial properly accounted for at its conclusion?** | **4. Were patients, health workers and study personnel ‘blind’ to treatment?** | **5. Were the groups similar at the start of the trial?** | **6. Aside from the experimental intervention, were the groups treated equally?** | **7. How large**  **was the treatment effect?** | **8. How precise was the estimate of the treatment effect?** | **9. Can the results be applied to the local population, or in your context?** | **10. Were all clinically important outcomes considered?** |
| --- | --- | --- | --- | --- | --- | --- | --- | --- | --- | --- |
| (31) Noome  etal 2016  (Netherlands)  Effectiveness of supporting intensive care units on implementing the guideline ‘End-of-life care in the intensive care unit, nursing care’: a cluster randomized controlled trial  **(7–8 Moderate high)** | **Yes**, the trial is clearly focused on the population studied (16 nurses participated in this RCT).  There is clear focus on the intervention given (the intervention was that one group received a  programme supporting the implementation  of the guidelines, and the other control group had to implement  the guidelines independently).  •The comparator is given.  There is clear focus on the outcomes considered.  An increase in adherence to the guidelines was found in both groups, as well as improvements and significant  differences. However, adherence to the guidelines was greater in the intervention group than in the control group. | **Yes,** the researcher explains the process of intervention as starting with education on implementation, strategies and project  management  coaching and support through the programme:  • The steps of Grol’s model  describing specific goals, analysis of the target group, current practice and context.  • Time to share experiences on the performance of different  steps of implementation.  • Workshops with themes introduced by implementation  leaders . | \| **Yes,** the researcher described how  three ICUs decided to cease their  participation in the study within reason,  and these ICUs were allocated to the control group (after randomization )In addition, one ICU ended their participation just before the last measurement,  due to severe illness  The trial was not stopped early, but not all the participants were analysed and accounted in the conclusion for different reasons. \| \| --- \| | \| **Cannot tell.** The researcher did not consider mentioning this point. This question may be inapplicable in this study \| \| --- \| | **Yes,** all the participants are nurses but other factors such as gender could affect the outcome. | **Yes.** | **Yes**, the researcher considered  the effectiveness of supporting ICU during the implementation.    The primary outcome is clear and specified scoring 25 recommendation regarding the guidelines.  The researcher explained the results in detail for each group.  The results showed that:  “increase in adherence to the guideline recommendations  was found in both groups, as well as improvements and significant  differences. However, the use of the guidelines was greater in the intervention group than the control group.” | **Yes,** in the results the researcher described the difference between the intervention and control group. For example, the intervention group shows an increase *in adherence to the guidelines of an interdisciplinary team* whereas the control group does not.  For *communication with patient and family* the control group scores significantly higher than the intervention group (P 0.001). | **Yes.** | **Yes,** the results show that the adherence to the guideline recommendation was greater in the intervention group. |

**A3: SURVEY (INCLUDING PRE-TEST PROBABILITIES) CHECKLIST**

| How do you rate this paper? 1 2 3 4 5 6 7 **8** 9 10 Rating scale: (1–2 Low) (3–4 Moderate low) (5–6 Moderate) **(7–8 Moderate high**) (9–10 High) |
| --- |

**Study title:** Barriers to End-of-Life Care in the Intensive Care Unit: Perceptions Vary by Level of Training, Discipline, and Institution (35)

| **1.0 OBJECTIVES AND HYPOTHESES** | |
| --- | --- |
| 1.1 Are the objectives of the study clearly stated? | Yes, it is clearly stated that the aim of the study is to determine obstacles to quality end-of-life (EOL) care provision in an intensive care unit (ICU), related to training and regulation. |
| **2.0 DESIGN** | |
| 2.1 Is the study design suitable for the objectives? | Yes |
| 2.2 Who/what was studied? | Who: teaching hospital professionals including residents, attendees, nurses and ICU fellows, across two locations  What: a questionnaire relating to obstacles to provision of quality EOL ICU care, from a training and regulation perspective |
| 2.3 Was this the right sample to answer the objectives? | An appropriate sample was selected to give responses: 125 residents, 20 fellows, 13 attendees, and 60 nurses. |
| **2.4 Did the subject represent the full spectrum of the population of interest?** | Yes, the full spectrum was represented, although weighted towards residents and nurses. |
| 2.5 Is the study large enough to achieve its objectives? Have sample size estimates been performed? | ICU trainee nurses, attendees, and physicians were invited to respond to the questionnaire. There was an adequate sample size, although the author carried out an estimates sample. |
| 2.6 Were all subjects accounted for? | Yes |
| 2.7 Were all appropriate outcomes considered? | Yes |
| 2.8 Has ethical approval been obtained if appropriate? | The study was approved by the New York University School of Medicine IRB (approval for 08-581). |
| **2.9 What measures were made to contact non-responders?** | This information was not stated in the study. |
| **2.10 What was the response rate?** | Response rate was 76% |
| **3.0 MEASUREMENT AND OBSERVATION** | |
| 3.1 Is it clear what was measured, how it was measured and what the outcomes were? | There are three areas covered by the questionnaire on potential EOL ICU care obstacles: factors related to the family of the patient, clinician factors, and institutional factors. One further area was expanded on by the researcher: training-based factors, to assess how educational training is prescribed. Notable variations according to training level were seen in the obstacles to EOL care described. |
| 3.2 Are the measurements valid? | A questionnaire tool to examine the obstacles to quality EOL care in an ICU was created and verified by the author. |
| 3.3 Are the measurements reliable? | This information is not included in the study. |
| 3.4 Are the measurements reproducible? | Yes |
| **4.0 PRESENTATION OF RESULTS** | |
| 4.1 Are the basic data adequately described? | Yes, the obtained data are presented textually and in tabular form. |
| 4.2 Are the results presented clearly, objectively and in sufficient detail to enable readers to make their own judgement? | Yes, the study includes clear and detailed findings. |
| 4.3 Are the results internally consistent, i.e. do the numbers add up properly? | Yes |
| **5.0 ANALYSIS** | |
| 5.1 Are the data suitable for analysis? | Yes |
| 5.2 Are the methods appropriate to the data? | Data analysis was performed using the statistical software Stata (version 10.0). |
| 5.3 Are any statistics correctly performed and interpreted? | Yes |
| **6.0 DISCUSSION** | |
| 6.1 Are the results discussed in relation to existing knowledge on the subject and study objectives? | Yes |
| 6.2 Is the discussion biased? | Certain limitations of the findings are detailed in the paper. The discussion is free of bias. |
| **6.3 Can the results be generalized?** | Yes |
| **7.0 INTERPRETATION** | |
| 7.1 Are the authors’ conclusions justified by the data? | Yes |
| 7.2 What level of evidence has this paper presented? (using CEBM levels) | Level 2 |
| 7.3 Does this paper help me answer my problem? | Yes, various obstacles to quality EOL care in an ICU are identified as outcomes of this research. |
| How do you rate this paper now? 1 2 3 4 5 6 7 **8** 9 10  In addition, answer the following questions with regards to local practice | |
| **8.0 IMPLEMENTATION** | |
| 8.1 Can any necessary change be implemented in practice? | Not applicable |
| 8.2 What aids to implementation exist? | Not applicable |
| 8.3 What barriers to implementation exist? | Not applicable |

**Study title:** Critical Care Nurses’ Perceptions of Obstacles, Support, and Knowledge Needed in Providing Quality End-of-Life Care (36)

| How do you rate this paper? 1 2 3 **4** 5 6 7 8 9 10 Rating scale (1–2 Low) **(3–4 Moderate low)** (5–6 Moderate) (7–8 Moderate high) (9–10 High) |
| --- |

| **1.0 OBJECTIVES AND HYPOTHESES** | |
| --- | --- |
| 1.1 Are the objectives of the study clearly stated? | Yes, the aims are clearly stated. The study aims to examine factors either encouraging or inhibiting quality EOL care in critical care environments.  1. What are critical care nurses’ perceptions of obstacles to, and support for, EOL care in a critical care environment?  2. What do critical care nurses perceive to be the required knowledge and skills to provide quality EOL care? |
| **2.0 DESIGN** |  |
| 2.1 Is the study design suitable for the objectives? | Yes |
| 2.2 Who/what was studied? | Who: critical care nurses  What: examination of the factors that either encourage or inhibit quality EOL care in critical care environments |
| 2.3 Was this the right sample to answer the objectives? | Yes, the selected participants were critical care nurses, which is appropriate for obtaining the factors they perceive as encouraging or inhibiting EOL care. |
| **2.4 Did the subject represent the full spectrum of the population of interest?** | Yes |
| 2.5 Is the study large enough to achieve its objectives? Have sample size estimates been performed? | The questionnaire was provided to a total of 180 of nurses, of whom 56 gave responses. There was no assessment of estimated sample size. |
| 2.6 Were all subjects accounted for? | Yes |
| 2.7 Were all appropriate outcomes considered? | Yes |
| 2.8 Has ethical approval been obtained if appropriate? | Yes, approval was obtained from the hospital institutional  review board. |
| **2.9 What measures were made to contact non-responders?** | The questionnaire was provided to all 180 nurses in critical care at the hospital using internal email, but no measures were taken to contact non-responders. |
| **2.10 What was the response rate?** | A total of 56 responses were received from 180 nurses, a low rate of 31%. |
| **3.0 MEASUREMENT AND OBSERVATION** | |
| 3.1 Is it clear what was measured, how it was measured and what the outcomes were? | Yes, the factors perceived by critical care nurses to either encourage or inhibit quality EOL care were recorded, from the perspectives of both barriers and educational requirements. |
| 3.2 Are the measurements valid? | The researcher developed the questionnaire. The study does not give any description of validation of the method. |
| 3.3 Are the measurements reliable? | The Beckstrand and Kirchhoff study recorded subscale internal consistency within 0.81–0.89. |
| 3.4 Are the measurements reproducible? | Not presented |
| **4.0 PRESENTATION OF RESULTS** | |
| 4.1 Are the basic data adequately described? | Yes, the description of the data is good. |
| 4.2 Are the results presented clearly, objectively and in sufficient detail to enable readers to make their own judgement? | Yes, the findings are clear and free of bias. They are adequate for readers to infer their own judgement. |
| 4.3 Are the results internally consistent, i.e. do the numbers add up properly? | Yes |
| **5.0 ANALYSIS** | |
| 5.1 Are the data suitable for analysis? | Yes |
| 5.2 Are the methods appropriate to the data? | Yes, analysis is performed by SPSS Statistical software. |
| 5.3 Are any statistics correctly performed and interpreted? | Yes, the interpretation of the findings is presented both textually and in tabular form. |
| **6.0 DISCUSSION** | |
| 6.1 Are the results discussed in relation to existing knowledge on the subject and study objectives? | Yes |
| 6.2 Is the discussion biased? | There is no bias present. |
| **6.3 Can the results be generalized?** | Yes, on the condition of a large sample. |
| **7.0 INTERPRETATION** | |
| 7.1 Are the authors’ conclusions justified by the data? | Yes, the researcher summarized the findings. |
| 7.2 What level of evidence has this paper presented? (using CEBM levels) | Level 1–2 |
| 7.3 Does this paper help me answer my problem? | Yes, obstacles to quality EOL patient care provision are ascertained. |
| How do you rate this paper now? 1 2 3 **4** 5 6 7 8 9 10  In addition, answer the following questions with regards to local practice | |
| **8.0 IMPLEMENTATION** | |
| 8.1 Can any necessary change be implemented in practice? | Not applicable |
| 8.2 What aids to implementation exist? | Not applicable |
| 8.3 What barriers to implementation exist? | Not applicable |

**Study title:** Current practices for withdrawal of life support in intensive care units (28)

| How do you rate this paper? 1 2 3 4 5 6 7 8 **9** 10 Rating scale: (1–2 Low) (3–4 Moderate low) (5–6 Moderate) (7–8 Moderate high) **(9–10 High)** |
| --- |

| **1.0 OBJECTIVES AND HYPOTHESES** | |
| --- | --- |
| 1.1 Are the objectives of the study clearly stated? | Yes, it is clearly stated that the study objective is to examine withdrawal of life support in US intensive care units with respect to the training, advice and other support given to nurses, the manner of their participation, and the subsequent process. |
| **2.0 DESIGN** | |
| 2.1 Is the study design suitable for the objectives? | Yes |
| 2.2 Who/what was studied? | Who: American Association of Critical Care Nurses registered nursing staff  What: Responses to a randomly distributed survey titled “Critical Care Nurses Participation in the Withdrawal of Life Support” |
| 2.3 Was this the right sample to answer the objectives? | Yes, the high number of survey participants is appropriate. |
| **2.4 Did the subject represent the full spectrum of the population of interest?** | Yes |
| 2.5 Is the study large enough to achieve its objectives? Have sample size estimates been performed? | The study size is relatively small for a descriptive study. Sample size estimates were carried out, giving a 50% estimated proportion of responses with error 3.1%. |
| 2.6 Were all subjects accounted for? | Yes |
| 2.7 Were all appropriate outcomes considered? | Yes |
| 2.8 Has ethical approval been obtained if appropriate? | Yes, the study was approved by the Health Sciences Minimal Risk Institutional Review Board of the University of Wisconsin–Madison, accompanied by a written consent waiver. |
| **2.9 What measures were made to contact non-responders?** | A coupon was offered by the researcher to participants as a form of compensation. |
| **2.10 What was the response rate?** | A rate of 48.4% responses was achieved. |
| **3.0 MEASUREMENT AND OBSERVATION** | |
| 3.1 Is it clear what was measured, how it was measured and what the outcomes were? | Yes, withdrawal of life support was examined with respect to the training, advice and other support given to nurses, via an author-devised questionnaire titled “Critical Care Nurses Participation in the Withdrawal of Life Support”. |
| 3.2 Are the measurements valid? | Yes, the author validated and piloted the survey. |
| 3.3 Are the measurements reliable? | Yes, there was an interrater reliability of 99%.  The handwritten responses were verified by Teleforms. |
| 3.4 Are the measurements reproducible? | Yes |
| **4.0 PRESENTATION OF RESULTS** | |
| 4.1 Are the basic data adequately described? | Yes, the textual and tabular descriptions of the data are clear. |
| 4.2 Are the results presented clearly, objectively and in sufficient detail to enable readers to make their own judgement? | Yes, detailed and easily interpreted results are presented by the author. |
| 4.3 Are the results internally consistent, i.e. do the numbers add up properly? | Yes |
| **5.0 ANALYSIS** | |
| 5.1 Are the data suitable for analysis? | Yes |
| 5.2 Are the methods appropriate to the data? | Yes |
| 5.3 Are any statistics correctly performed and interpreted? | Yes, using SPSS Statistical software (v16.0, SPSS Inc, Chicago, IL). |
| **6.0 DISCUSSION** | |
| 6.1 Are the results discussed in relation to existing knowledge on the subject and study objectives? | Yes, the author gives consideration to existing knowledge in their discussion. |
| 6.2 Is the discussion biased? | There is no bias present. |
| **6.3 Can the results be generalized?** | Yes |
| **7.0 INTERPRETATION** | |
| 7.1 Are the authors’ conclusions justified by the data? | Yes |
| 7.2 What level of evidence has this paper presented? (using CEBM levels) | Can’t tell |
| 7.3 Does this paper help me answer my problem? | Yes, the issues of the support and obstacles to EOL care provision are presented. |
| How do you rate this paper now? 1 2 3 4 5 6 7 8 **9** 10  In addition, answer the following questions with regards to local practice | |
| **8.0 IMPLEMENTATION** | |
| 8.1 Can any necessary change be implemented in practice? | Not applicable |
| 8.2 What aids to implementation exist? | Not applicable |
| 8.3 What barriers to implementation exist? | Not applicable |

**Study title:** Limiting life-sustaining treatment in German intensive care units: A multi-professional survey (29)

| How do you rate this paper? 1 2 3 4 5 **6** 7 8 9 10 Rating scale (1–2 Low) (3–4 Moderate low) **(5–6 Moderate)** (7–8 Moderate high) (9–10 High). |
| --- |

| **1.0 OBJECTIVES AND HYPOTHESES** | |
| --- | --- |
| 1.1 Are the objectives of the study clearly stated? | Yes, the aim is clearly stated: to examine opinions and practice on the limits to LST from the viewpoints of German physicians and nurses working in intensive care. |
| **2.0 DESIGN** | |
| 2.1 Is the study design suitable for the objectives? | Yes |
| 2.2 Who/what was studied? | A survey was devised to gather responses from nurses and physicians regarding their opinions and experiences of decision making in EOL care. The survey included the German law definition of limits to LST and combinations of treatment withdrawal and limitation. |
| 2.3 Was this the right sample to answer the objectives? | Yes, the sample selection matches the research aim. |
| **2.4 Did the subject represent the full spectrum of the population of interest?** | Yes, ICU nurses and physicians were included as the survey participants. |
| 2.5 Is the study large enough to achieve its objectives? Have sample size estimates been performed? | The size of the sample appears relatively low. The study did not include sample size estimates. |
| 2.6 Were all subjects accounted for? | Yes |
| 2.7 Were all appropriate outcomes considered? | Yes, although missing data was omitted by the author. |
| 2.8 Has ethical approval been obtained if appropriate? | Yes, the hospital ethics committee accepted the study. |
| **2.9 What measures were made to contact non-responders?** | The manager and head of the unit shared the survey with participants. No action was taken regarding non-responders. |
| **2.10 What was the response rate?** | The response rates were 56% and 51% of nurses and physicians, respectively. |
| **3.0 MEASUREMENT AND OBSERVATION** | |
| 3.1 Is it clear what was measured, how it was measured and what the outcomes were? | Yes |
| 3.2 Are the measurements valid? | Not presented in this study |
| 3.3 Are the measurements reliable? | Not presented in this study |
| 3.4 Are the measurements reproducible? | Not presented in this study |
| **4.0 PRESENTATION OF RESULTS** | |
| 4.1 Are the basic data adequately described? | Yes, the data were adequately described textually. |
| 4.2 Are the results presented clearly, objectively and in sufficient detail to enable readers to make their own judgement? | Yes, although some questions were not portrayed in tabular or graphical form. |
| 4.3 Are the results internally consistent, i.e. do the numbers add up properly? | Yes |
| **5.0 ANALYSIS** | |
| 5.1 Are the data suitable for analysis? | Yes |
| 5.2 Are the methods appropriate to the data? | Yes, SPSS was used. |
| 5.3 Are any statistics correctly performed and interpreted? | Yes, some statistics were displayed in figures or tables. |
| **6.0 DISCUSSION** | |
| 6.1 Are the results discussed in relation to existing knowledge on the subject and study objectives? | Yes, due consideration is given to recent related literature. |
| 6.2 Is the discussion biased? | No bias was present in this section. |
| **6.3 Can the results be generalized?** | Yes, it is possible to generalize the result of this study |
| **7.0 INTERPRETATION** | |
| 7.1 Are the authors’ conclusions justified by the data? | Yes |
| 7.2 What level of evidence has this paper presented? (using CEBM levels) | Can’t tell |
| 7.3 Does this paper help me answer my problem? | Yes, the paper addresses the approach and challenges of ICU physicians and nurses regarding EOL care. |
| How do you rate this paper now? 1 2 3 4 5 6 7 8 9 10  In addition, answer the following questions with regards to local practice | |
| **8.0 IMPLEMENTATION** | |
| 8.1 Can any necessary change be implemented in practice? | Not applicable |
| 8.2 What aids to implementation exist? | Not applicable |
| 8.3 What barriers to implementation exist? | Not applicable |

**Study title:** Palliative Care in the Intensive Care Unit: Are Residents Well Trained to Provide Optimal Care to Critically ill Patients (19)

| How do you rate this paper? 1 **2** 3 4 5 6 7 8 9 10 Rating scale **(1–2 Low)** (3–4 Moderate low) (5–6 Moderate) (7–8 Moderate high) (9–10 High) |
| --- |

| **1.0 OBJECTIVES AND HYPOTHESES** | |
| --- | --- |
| 1.1 Are the objectives of the study clearly stated? | Yes, there is a clearly stated objective of assessing the knowledge of residents regarding use of PC EOL care in a tertiary hospital ICU, as well as any obstacles they see to this. |
| **2.0 DESIGN** | |
| 2.1 Is the study design suitable for the objectives? | Yes |
| 2.2 Who/what was studied? | Who: residents at the tertiary hospital  What: A questionnaire on the knowledge and skillset of residents, and obstacles they see to ICU palliative care |
| 2.3 Was this the right sample to answer the objectives? | Yes |
| **2.4 Did the subject represent the full spectrum of the population of interest?** | Yes |
| 2.5 Is the study large enough to achieve its objectives? Have sample size estimates been performed? | The study sample size was just 30, which is insufficient. |
| 2.6 Were all subjects accounted for? | Yes |
| 2.7 Were all appropriate outcomes considered? | Yes |
| 2.8 Has ethical approval been obtained if appropriate? | Yes, the St Louis University Institutional Review Board accepted it. |
| **2.9 What measures were made to contact non-responders?** | There are no measures described in the study. |
| **2.10 What was the response rate?** | The response rate was 38.46%. |
| **3.0 MEASUREMENT AND OBSERVATION** | |
| 3.1 Is it clear what was measured, how it was measured and what the outcomes were? | Yes, this information is described clearly in the paper. |
| 3.2 Are the measurements valid? | The questionnaire was developed on the basis of a literature review the researcher conducted into ICU PC and EOL care, with particular emphasis on the training and knowledge of residents. However, the study did not test the validity of the questions. |
| 3.3 Are the measurements reliable? | Not presented in this paper |
| 3.4 Are the measurements reproducible? | Not presented in this paper |
| **4.0 PRESENTATION OF RESULTS** | |
| 4.1 Are the basic data adequately described? | Yes, adequate description is given by the author. |
| 4.2 Are the results presented clearly, objectively and in sufficient detail to enable readers to make their own judgement? | Yes, there is a good level of detail and clarity to the results. However, this section of the paper is of insufficient length. |
| 4.3 Are the results internally consistent, i.e. do the numbers add up properly? | Yes |
| **5.0 ANALYSIS** | |
| 5.1 Are the data suitable for analysis? | Yes |
| 5.2 Are the methods appropriate to the data? | Yes; however, this is not presented in the study. |
| 5.3 Are any statistics correctly performed and interpreted? | Yes |
| **6.0 DISCUSSION** | |
| 6.1 Are the results discussed in relation to existing knowledge on the subject and study objectives? | Yes, appropriate discussion is held on the context of the results within existing literature. |
| 6.2 Is the discussion biased? | The discussion is not biased. |
| **6.3 Can the results be generalized?** | No, because of the small sample size. |
| **7.0 INTERPRETATION** | |
| 7.1 Are the authors’ conclusions justified by the data? | Yes |
| 7.2 What level of evidence has this paper presented? (using CEBM levels) | Can’t tell |
| 7.3 Does this paper help me answer my problem? | Yes, the findings addressed the investigation into obstacles to EOL provision. |
| **8.0 IMPLEMENTATION** | |
| 8.1 Can any necessary change be implemented in practice? | Not applicable |
| 8.2 What aids to implementation exist? | Not applicable |
| 8.3 What barriers to implementation exist? | Not applicable |

**Study title:** EfCCNa survey: European intensive care nurses’ attitudes and beliefs towards end‐of‐life care (38)

| How do you rate this paper? 1 2 3 **4** 5 6 7 8 9 10 Rating scale (1–2 Low) **(3–4 Moderate low)** (5–6 Moderate) (7–8 Moderate high) (9–10 High) |
| --- |

| **1.0 OBJECTIVES AND HYPOTHESES** | |
| --- | --- |
| 1.1 Are the objectives of the study clearly stated? | Yes, the abstract and introduction sections clearly state the objective of assessing the experiences and opinions towards EOL care of intensive care nurses in Europe. |
| **2.0 DESIGN** | |
| 2.1 Is the study design suitable for the objectives? | Yes |
| 2.2 Who/what was studied? | Who: nurse attendees of the second European federation of  Critical Care Nursing associations (EfCCNa) conference on critical care nursing in Europe, hosted in November 2005 in Amsterdam, Netherlands.  What: the experiences and opinions towards EOL care of intensive care nurses in Europe. |
| 2.3 Was this the right sample to answer the objectives? | Yes |
| **2.4 Did the subject represent the full spectrum of the population of interest?** | Yes, they were exclusively European critical care nurses. Those from nations outside Europe were not included. |
| 2.5 Is the study large enough to achieve its objectives? Have sample size estimates been performed? | There appears to be a large sample, although there were no sample size estimates taken. The author notes that a return of 162 responses out of 419 was less than the rate anticipated from conference attendees. |
| 2.6 Were all subjects accounted for? | Yes |
| 2.7 Were all appropriate outcomes considered? | Yes |
| 2.8 Has ethical approval been obtained if appropriate? | Yes, approval was granted by six global EfCCNa Scientific Committee members, who confirmed that the ethical research conduct requirements were met by the study. |
| **2.9 What measures were made to contact non-responders?** | Conference delegates encouraged all the participants to take part in the survey. |
| **2.10 What was the response rate?** | There was a 39% response rate. |
| **3.0 MEASUREMENT AND OBSERVATION** | |
| 3.1 Is it clear what was measured, how it was measured and what the outcomes were? | Yes, the approaches to EOL intensive care by European critical nurses was clearly measured. |
| 3.2 Are the measurements valid? | Yes, the validity of the survey content was tested in three pilot studies in different Australian states with a sample size of 50 nurses in intensive care. |
| 3.3 Are the measurements reliable? | Yes, the measurements are reliable. This was also verified somewhat by the process of the pilot studies, which included feedback from participants on any unclear phrasing in the questions. |
| 3.4 Are the measurements reproducible? | Yes, the questionnaire is transferable to other populations. |
| **4.0 PRESENTATION OF RESULTS** | |
| 4.1 Are the basic data adequately described? | Yes |
| 4.2 Are the results presented clearly, objectively and in sufficient detail to enable readers to make their own judgement? | Yes, clear findings are portrayed both textually and in tabular form. |
| 4.3 Are the results internally consistent, i.e. do the numbers add up properly? | Yes |
| **5.0 ANALYSIS** | |
| 5.1 Are the data suitable for analysis? | Yes |
| 5.2 Are the methods appropriate to the data? | Yes, SPSS was employed by the author. |
| 5.3 Are any statistics correctly performed and interpreted? | Yes |
| **6.0 DISCUSSION** | |
| 6.1 Are the results discussed in relation to existing knowledge on the subject and study objectives? | Yes, adequate discussion is given on the context of the results within recent literature. |
| 6.2 Is the discussion biased? | My interpretation of this section was that no bias was present on behalf of the author. |
| **6.3 Can the results be generalized?** | Yes, on the condition that it is constrained to a similar population and setting. |
| **7.0 INTERPRETATION** | |
| 7.1 Are the authors’ conclusions justified by the data? | Yes, there is consideration given to opinions informed by ethics or religion that can be factors in making decisions in this context. |
| 7.2 What level of evidence has this paper presented? (using CEBM levels) | Level 1–2 |
| 7.3 Does this paper help me answer my problem? | Yes, this study discussed some of the factors associated with attitude regarding EOL care. |
| How do you rate this paper now? 1 2 3 4 5 6 7 **8** 9 10  In addition, answer the following questions with regards to local practice | |
| **8.0 IMPLEMENTATION** | |
| 8.1 Can any necessary change be implemented in practice? | Not applicable |
| 8.2 What aids to implementation exist? | Not applicable |
| 8.3 What barriers to implementation exist? | Not applicable |

**Study title:** Policies of withholding and withdrawal of life-sustaining treatment in critically ill patients on cardiac intensive

care units in Germany: a national survey (42)

| How do you rate this paper? 1 **2** 3 4 5 6 7 8 9 10 Rating scale **(1–2 Low)** (3–4 Moderate low) (5–6 Moderate) (7–8 Moderate high) (9–10 High) |
| --- |

| **1.0 OBJECTIVES AND HYPOTHESES** | |
| --- | --- |
| 1.1 Are the objectives of the study clearly stated? | Yes, there is a clear aim to investigate the process in Germany regarding decisions to withhold and/or withdraw (WH/WD) cardiac ICU life support treatment. |
| **2.0 DESIGN** | |
| 2.1 Is the study design suitable for the objectives? | Yes |
| 2.2 Who/what was studied? | Who: ICU clinical directors, senior physicians and head nurses  What: a 22-question survey (16 medical, 6 ethical) was shared on the topic of WH/WD of life support treatment |
| 2.3 Was this the right sample to answer the objectives? | Yes |
| **2.4 Did the subject represent the full spectrum of the population of interest?** | Yes |
| 2.5 --Is the study large enough to achieve its objectives? Have sample size estimates been performed? | The sample size was 86, but no sample size estimates were carried out. |
| 2.6 Were all subjects accounted for? | Yes |
| 2.7 Were all appropriate outcomes considered? | Yes |
| 2.8 Has ethical approval been obtained if appropriate? | This is not presented in the paper. |
| **2.9 What measures were made to contact non-responders?** | Not presented |
| **2.10 What was the response rate?** | The response rate was 36%. |
| **3.0 MEASUREMENT AND OBSERVATION** | |
| 3.1 Is it clear what was measured, how it was measured and what the outcomes were? | Yes, measurement of the medical factors in decision making around WH/WD of life support treatment was carried out using a survey specially designed by the author, containing 22 questions (16 medical, 6 ethical).The factors assessed comprised age of elderly patients and low anticipated standards of life. |
| 3.2 Are the measurements valid? | This is not addressed in the paper. |
| 3.3 Are the measurements reliable? | This is not addressed in the paper. |
| 3.4 Are the measurements reproducible? | Yes |
| **4.0 PRESENTATION OF RESULTS** | |
| 4.1 Are the basic data adequately described? | Yes, there is adequate textual and tabular description of the data. However, in my opinion the results section is insufficient in length. |
| 4.2 Are the results presented clearly, objectively and in sufficient detail to enable readers to make their own judgement? | The findings are clear but inadequately detailed. In my opinion the results section is insufficient in length. |
| 4.3 Are the results internally consistent, i.e. do the numbers add up properly? | Yes |
| **5.0 ANALYSIS** | |
| 5.1 Are the data suitable for analysis? | Yes |
| 5.2 Are the methods appropriate to the data? | The researcher employed R (version 2.12.1) open-source software |
| 5.3 Are any statistics correctly performed and interpreted? | Yes, the statistics are interpreted both textually and in tabular form. |
| **6.0 DISCUSSION** | |
| 6.1 Are the results discussed in relation to existing knowledge on the subject and study objectives? | Yes, due consideration is given to the relation of the findings to wider subject knowledge. |
| 6.2 Is the discussion biased? | No bias is present in this section. |
| **6.3 Can the results be generalized?** | Yes |
| **7.0 INTERPRETATION** | |
| 7.1 Are the authors’ conclusions justified by the data? | Yes |
| 7.2 What level of evidence has this paper presented? (using CEBM levels) | Can’t tell |
| 7.3 Does this paper help me answer my problem? | Yes, the paper addresses various issues that affect palliative care. |
| How do you rate this paper now? 1 **2** 3 4 5 6 7 8 9 10  In addition, answer the following questions with regards to local practice | |
| **8.0 IMPLEMENTATION** | |
| 8.1 Can any necessary change be implemented in practice? | Not applicable |
| 8.2 What aids to implementation exist? | Not applicable |
| 8.3 What barriers to implementation exist? | Not applicable |

**Study title:** Characteristics and Outcomes of Ethics Consultations in an Oncologic Intensive Care Unit (41)

| How do you rate this paper? 1 2 3 4 **5** 6 7 8 9 10 Rating scale (1–2 Low) (3–4 Moderate low) **(5–6 Moderate)** (7–8 Moderate high) (9–10 High) |
| --- |

| **1.0 OBJECTIVES AND HYPOTHESES** | |
| --- | --- |
| 1.1 Are the objectives of the study clearly stated? | Yes, there is a clearly stated objective of assessing the frequency, features, and results of ethics consultations regarding cancer patients in critical care. |
| **2.0 DESIGN** | |
| 2.1 Is the study design suitable for the objectives? | Yes, the study is suitably designed. |
| 2.2 Who/what was studied? | Who: the consultants who compiled the ethical consultation record being examined and are responsible for training nurses, social workers, psychiatrists, surgeons, and intensivists in medical ethics.  What: A past ethical consultation that was run in ICU staff regarding adult inpatients of a 20-berth medical–surgical ICU located in the Memorial Sloan Kettering Cancer Center (MSKCC). |
| 2.3 Was this the right sample to answer the objectives? | Yes, an appropriate sample is selected for the study aim. |
| **2.4 Did the subject represent the full spectrum of the population of interest?** | Yes |
| 2.5 Is the study large enough to achieve its objectives? Have sample size estimates been performed? | There were 53 places in the ethics consultation (26%) pertaining to 5010 patient arrivals in the ICU. |
| 2.6 Were all subjects accounted for? | Yes |
| 2.7 Were all appropriate outcomes considered? | Yes |
| 2.8 Has ethical approval been obtained if appropriate? | The institutional review board approved the study. |
| **2.9 What measures were made to contact non-responders?** | Not applicable |
| **2.10 What was the response rate?** | Not applicable |
| **3.0 MEASUREMENT AND OBSERVATION** | |
| 3.1 Is it clear what was measured, how it was measured and what the outcomes were? | Yes, the ethics consultations were being measured, including the clinical history of the consultant (such as their job and length of time in ethics) and the nature of the staff asking for the consultation (ICU staff, clinical staff outside the ICU, patients, or surrogates). The capability of a patient to make decisions was assessed (on the basis of a current ICU attending physician assessment), as well as advance directive and/or healthcare professional or surrogate presence, and their resuscitation condition before and after the consultation. |
| 3.2 Are the measurements valid? | This was not addressed in the study. |
| 3.3 Are the measurements reliable? | This was not addressed in the study. |
| 3.4 Are the measurements reproducible? | Yes |
| **4.0 PRESENTATION OF RESULTS** | |
| 4.1 Are the basic data adequately described? | Yes, the data is well described both textually and in tabular form. |
| 4.2 Are the results presented clearly, objectively and in sufficient detail to enable readers to make their own judgement? | Yes |
| 4.3 Are the results internally consistent, i.e. do the numbers add up properly? | Yes |
| **5.0 ANALYSIS** | |
| 5.1 Are the data suitable for analysis? | Yes |
| 5.2 Are the methods appropriate to the data? | Yes, the method of descriptive statistics was applied to the data obtained, via Microsoft Excel 2010 (Redmond, Washington). |
| 5.3 Are any statistics correctly performed and interpreted? | Yes |
| **6.0 DISCUSSION** | |
| 6.1 Are the results discussed in relation to existing knowledge on the subject and study objectives? | Yes, adequate discussion was given on the relation of the findings to wider subject and objective knowledge. |
| 6.2 Is the discussion biased? | The limitations on bias in this section were discussed. |
| **6.3 Can the results be generalized?** | Yes |
| **7.0 INTERPRETATION** | |
| 7.1 Are the authors’ conclusions justified by the data? | Yes |
| 7.2 What level of evidence has this paper presented? (using CEBM levels) | Level 3 |
| 7.3 Does this paper help me answer my problem? | Yes, the aim of assessing the ICU patient ethical consultation from a healthcare organization was addressed. The consultation in question is portrayed as an ICU facility. |
| How do you rate this paper now? 1 2 3 4 5 6 7 **8** 9 1  In addition, answer the following questions with regards to local practice | |
| **8.0 IMPLEMENTATION** | |
| 8.1 Can any necessary change be implemented in practice? | Not applicable |
| 8.2 What aids to implementation exist? | Not applicable |
| 8.3 What barriers to implementation exist? | Not applicable |

**A4: Mixed Methods Appraisal Tool (MMAT) – Version 2011**

**PART I. MMAT criteria & one-page template (to be included in appraisal forms)**

| **Types of mixed methods**  **study components or**  **primary studies** | **Methodological quality criteria (see tutorial for definitions and examples) responses** | **Response** | |  | |
| --- | --- | --- | --- | --- | --- |
|  |  | **Yes** | **No** | **Can’t tell** | **Comments** |
|  | **Title:** A pilot audit of the process of end-of-life decision-making in the intensive care unit (40) | | | | |
| **Screening questions**  **(for all types)** | Are there clear qualitative and quantitative research questions (or objectives*), or a clear mixed methods question (or objective*)?   Do the collected data address the research question (objective)? E.g., consider whether the follow-up period is long enough for the  outcome to occur (for longitudinal studies or study components). | Yes, the objectives are presented clearly. This pilot audit was developed with two objectives: to collect informative ICU practice data; and to collect knowledge to form the basis of a quality assessment auditing tool. In particular, the author aimed to obtain solutions to these questions:  • Is it possible to obtain significant information regarding the factors proposed as comprising a best-practice way of dealing with withdrawal of treatment?  • Can a useful, continuous auditing tool be compiled?  The author held a structured interview with the intensivist responsible for recording the choice to withdraw treatment up to a week after this choice was made. The purpose of this was to learn about the attitudes to EOL decision making held by intensivists. |  |  |  |
| **5. Mixed methods**  Sequential explanatory design | 5.1. Is the mixed methods research design relevant to address the qualitative and quantitative research questions (or objectives), or the  qualitative and quantitative aspects of the mixed methods question (or objective)? | The justification for combining qualitative and quantitative approaches to address the research objectives is insufficiently described. |  |  |  |
|  | 5.2. Is the integration of qualitative and quantitative data (or results*) relevant to address the research question (objective)? | **Yes,** the research objectives are addressed and the findings are explained in relation to the combination of methods to provide a whole overview. That said, the section is insufficient in length and should be made more concise. |  |  |  |
|  | 5.3. Is appropriate consideration given to the limitations associated with this integration, e.g., the divergence of qualitative and quantitative  data (or results*) in a triangulation design? | Yes, the author describes some limitations in the paper.  The findings are presented using a descriptive method.  Data from recruited patients was gathered retrospectively. This may have led to less reliable response data.  The inclusion of an interview in the study facilitated collection of data on the viewpoints of intensivists, which an audit of solely medical records would not provide. |  |  |  |
| **Rating scale:** (1–2 Low) (3–4 Moderate low) **(5–6 Moderate)** (7–8 Moderate high) (9–10 High) | |  | | | |

**PART I. MMAT criteria & one-page template (to be included in appraisal forms)**

| **Types of mixed methods**  **study components or**  **primary studies** | **Methodological quality criteria (see tutorial for definitions and examples) responses** | **Response** | |  | |
| --- | --- | --- | --- | --- | --- |
|  |  | **Yes** | **No** | **Can’t tell** | **Comments** |
|  | **Title:** Improving ICU-Based Palliative Care Delivery: A Multicentre, Multidisciplinary Survey of Critical Care Clinician Attitudes and Beliefs (30) | | | | |
| **Screening questions**  **(for all types)** | Are there clear qualitative and quantitative research questions (or objectives*), or a clear mixed methods question (or objective*)?   Do the collected data allow address the research question (objective)? E.g., consider whether the follow-up period is long enough for the  outcome to occur (for longitudinal studies or study components). | The aims of the study are clearly stated: to investigate the uncertainty surrounding adequate collaborative models of general and specialist ICU palliative care, and the limitations on quality this causes. To this end, the approaches of nurses and physicians towards ICU palliative care provision were obtained by the author. |  |  |  |
| **5. Mixed methods**  Sequential explanatory design | 5.1. Is the mixed methods research design relevant to address the qualitative and quantitative research questions (or objectives), or the  qualitative and quantitative aspects of the mixed methods question (or objective)? | **Yes,** the author employed a cross-sectional investigation during May–November 2015, utilizing open-ended questionnaires encouraging participants to share their perspectives on preferable approaches to palliative care in ICUs, screening approaches to ascertain the right patients to receive specialist consultation, and personal triggers. |  |  |  |
|  | 5.2. Is the integration of qualitative and quantitative data (or results*) relevant to address the research question (objective)? | **Yes,** the study objectives are addressed by the researcher and the findings are explained in relation to the combination of methods and the wider perspective they provide. That said, the results appear separated to me; for instance, the qualitative findings and the survey findings are each described in individual paragraphs and the integration is not evident. |  |  |  |
|  | 5.3. Is appropriate consideration given to the limitations associated with this integration, e.g., the divergence of qualitative and quantitative  data (or results*) in a triangulation design? |  | There is discussion of certain limitations, although their link to the study aims or combination of methods is not evident. |  |  |
| **Rating scale: (1–2 Low) (**3–4 Moderate low) (5–6 Moderate) (7–8 Moderate high) (9–10 High) | |  | | | |

**PART I. MMAT criteria & one-page template (to be included in appraisal forms)**

| **Types of mixed methods**  **study components or**  **primary studies** | **Methodological quality criteria (see tutorial for definitions and examples) responses** | **Response** | |  | |
| --- | --- | --- | --- | --- | --- |
|  |  | **Yes** | **No** | **Can’t tell** | **Comments** |
|  | **Title:** Respecting the wishes of patients in intensive care units (37) | | | | |
| **Screening questions**  **(for all types)**  Sequential explanatory design | Are there clear qualitative and quantitative research questions (or objectives*), or a clear mixed methods question (or objective*)?   Do the collected data allow address the research question (objective)? E.g., consider whether the follow-up period is long enough for the outcome to occur (for longitudinal studies or study components). | Yes, there are clearly stated objectives: to investigate the reasons behind challenges to ICU nurses in respecting the desires of EOL care patients in Japan.  The researcher employed a survey of university hospital ICU nurses in Japan. This included an open-ended question inviting participants to share their opinions on why the desires of patients were disregarded. |  |  | The study does not state the period over which it was conducted. |
| **5. Mixed methods** | 5.1. Is the mixed methods research design relevant to address the qualitative and quantitative research questions (or objectives), or the  qualitative and quantitative aspects of the mixed methods question (or objective)? | **Yes,** the method is appropriate to the objectives, although in my opinion this research could be carried out solely qualitatively. |  |  |  |
|  | 5.2. Is the integration of qualitative and quantitative data (or results*) relevant to address the research question (objective)? | **Yes,** it is appropriate to combine qualitative and quantitative data, although the objectives of the study and discussion of findings are addressed only from a qualitative viewpoint by the researcher. |  |  | The results and discussion sections emphasize the qualitative findings. |
|  | 5.3. Is appropriate consideration given to the limitations associated with this integration, e.g., the divergence of qualitative and quantitative  data (or results*) in a triangulation design? | **No,** limitations are not discussed in the paper. |  |  |  |
| **Rating scale:** (1–2 Low) **(3–4 Moderate low)** (5–6 Moderate) (7–8 Moderate high) (9–10 High) | |  | | | |

**PART I. MMAT criteria & one-page template (to be included in appraisal forms)**

| **Types of mixed methods**  **study components or**  **primary studies** | **Methodological quality criteria (see tutorial for definitions and examples) responses** | **Response** | |  | |
| --- | --- | --- | --- | --- | --- |
|  |  | **Yes** | **No** | **Can’t tell** | **Comments** |
|  | **Title:** Resident reflections on end-of-life education: a mixed-methods study of the 3 Wishes (24) | | | | |
| **Screening questions**  **(for all types)**  Sequential explanatory design | Are there clear qualitative and quantitative research questions (or objectives*), or a clear mixed methods question (or objective*)?   Do the collected data allow address the research question (objective)? E.g., consider whether the follow-up period is long enough for the outcome to occur (for longitudinal studies or study components). | The aims of the study were clearly stated: to obtain the experiences of EOL-trained residents in an ICU rotation, and to ascertain the potential effects of the 3 Wishes Project.  *This involves adopting three wishes by the patient, their family or the clinicians, with the aim of aiding in dignifying the death of the patient, appreciating their life, and practically employing humanism.  The study was carried out via semi-structured interviews with a minimum of one relative or the patient, and between one and three clinicians. The purpose of these was to learn about the effect of the 3 Wishes Project on their end-of-life process experiences and general perspectives. |  |  | The study does not state the period over which it was conducted. |
| **5. Mixed methods** | 5.1. Is the mixed methods research design relevant to address the qualitative and quantitative research questions (or objectives), or the  qualitative and quantitative aspects of the mixed methods question (or objective)? | **Yes,** although the aims could have been achieved solely via qualitative methods. |  |  |  |
|  | 5.2. Is the integration of qualitative and quantitative data (or results*) relevant to address the research question (objective)? | **No,** the study aims and findings are addressed solely from a qualitative viewpoint. |  |  | The results and discussion sections emphasize the qualitative findings. |
|  | 5.3. Is appropriate consideration given to the limitations associated with this integration, e.g., the divergence of qualitative and quantitative  data (or results*) in a triangulation design? | **Yes,** the paper discusses certain limitations, although these are not linked to the integration of methods. |  |  |  |
| **Rating scale: (1–2 Low)** (3–4 Moderate low) (5–6 Moderate) (7–8 Moderate high) (9–10 High) | |  | | | |

**PART I. MMAT criteria & one-page template (to be included in appraisal forms)**

| **Types of mixed methods**  **study components or**  **primary studies** | **Methodological quality criteria (see tutorial for definitions and examples) responses** | **Response** | |  | |
| --- | --- | --- | --- | --- | --- |
|  |  | **Yes** | **No** | **Can’t tell** | **Comments** |
|  | **Title**: Nurses' perceptions of end-of-life care after multiple interventions for improvement (22) | | | | |
| **Screening questions**  **(for all types)**  Sequential explanatory design | Are there clear qualitative and quantitative research questions (or objectives*), or a clear mixed methods question (or objective*)?   Do the collected data allow address the research question (objective)? E.g., consider whether the follow-up period is long enough for the outcome to occur (for longitudinal studies or study components). | The research objectives were clearly stated: to investigate the opinions of nurses on (1) knowledge and skills, (2) working environment, (3) resources available to staff, (4) resources available to patients and relatives, and (5) work-related stress regarding EOL care pre- (phase 1) and post- (phase 2) application of improvements to the care. These improvements comprised a bereavement programme for relatives created by nurses, access to a palliative medicine and comfort care team, instructions printed in advance regarding withdrawing life-support treatment, employment of a clinical nurse specializing in mental health, and employee training in EOL care. |  |  |  |
| **5. Mixed methods** | 5.1. Is the mixed methods research design relevant to address the qualitative and quantitative research questions (or objectives), or the qualitative and quantitative aspects of the mixed methods question (or objective)? | **Yes** |  |  |  |
|  | 5.2. Is the integration of qualitative and quantitative data (or results*) relevant to address the research question (objective)? | **No,** the study objectives and findings are addressed in relation to both qualitative and quantitative information. |  |  |  |
|  | 5.3. Is appropriate consideration given to the limitations associated with this integration, e.g., the divergence of qualitative and quantitative data (or results*) in a triangulation design? | Yes, discussion is given on the limitations of the research; for instance, additional verification was needed for the investigator-developed tool. Despite early evidence that the study is internally consistent, additional validation methods should be employed to confirm reliability. |  |  |  |
| **Rating scale:** (1–2 Low) (3–4 Moderate low) **(5–6 Moderate**) (7–8 Moderate high) (9–10 High) | |  | | | |

**PART I. MMAT criteria & one-page template (to be included in appraisal forms)**

| **Types of mixed methods**  **study components or**  **primary studies** | **Methodological quality criteria (see tutorial for definitions and examples) responses** | **Response** | |  | |
| --- | --- | --- | --- | --- | --- |
|  |  | **Yes** | **No** | **Can’t tell** | **Comments** |
|  | **Title**: Palliative care professional development for critical care nurses: a multicentre programme (32) | | | | |
| **Screening questions**  **(for all types)**  Sequential explanatory design | Are there clear qualitative and quantitative research questions (or objectives*), or a clear mixed methods question (or objective*)?   Do the collected data allow address the research question (objective)? E.g., consider whether the follow-up period is long enough for the outcome to occur (for longitudinal studies or study components). | Yes, there is a clearly stated objective: to employ and assess a programme of professional development for ICU bedside nurses in EOL care. |  |  |  |
| **5. Mixed methods** | 5.1. Is the mixed methods research design relevant to address the qualitative and quantitative research questions (or objectives), or the qualitative and quantitative aspects of the mixed methods question (or objective)? | Yes, the paper addresses this clearly. |  |  |  |
|  | 5.2. Is the integration of qualitative and quantitative data (or results*) relevant to address the research question (objective)? | Yes |  |  |  |
|  | 5.3. Is appropriate consideration given to the limitations associated with this integration, e.g., the divergence of qualitative and quantitative data (or results*) in a triangulation design? | No limitations regarding the combination of qualitative and quantitative information are discussed. |  |  |  |
| **Rating scale:** (1–2 Low) (3–4 Moderate low) **(5–6 Moderate)** (7–8 Moderate high) (9–10 High) | |  | | | |
